# Safety and efficacy of Xiaoyao-san for the treatment of functional dyspepsia: a systematic review and meta-analysis of randomized controlled trials

**DOI:** 10.3389/fphar.2023.1114222

**Published:** 2023-04-12

**Authors:** Na-Yeon Ha, Hanul Lee, Haein Jeong, Seok-Jae Ko, Jae-Woo Park, Jinsung Kim

**Affiliations:** ^1^ Division of Digestive Diseases, Department of Internal Korean Medicine, Kyung Hee University Medical Center, Seoul, Republic of Korea; ^2^ Department of Clinical Korean Medicine, Graduate School, Kyung Hee University, Seoul, Republic of Korea; ^3^ Department of Gastroenterology, Kyung Hee University College of Korean Medicine, Kyung Hee University Hospital at Gangdong, Seoul, Republic of Korea; ^4^ Department of Gastroenterology, Kyung Hee University College of Korean Medicine, Kyung Hee University Medical Center, Seoul, Republic of Korea

**Keywords:** functional dyspepsia, Soyo-san, Xiaoyao-san, herbal medicine, systematic review, meta-analysis

## Abstract

**Objective:** Although *Xiaoyao-san* (XYS) is a popular herbal remedy for indigestion, there is insufficient evidence to recommend it as a treatment option for functional dyspepsia (FD). This review aimed to assess the safety and efficacy of XYS in patients with FD, compared to conventional Western medicine (WM).

**Methods:** Two independent reviewers searched for randomized controlled trials (RCTs) using 11 electronic databases, including Medline and Embase, to evaluate therapeutic effects of XYS on FD up to 31 January 2023. The primary outcome was the total clinical efficacy rate (TCE), and secondary outcomes included scores of dyspepsia-related symptoms (DSS) and incidence of adverse events (AEs). The risk of bias was evaluated using the Cochrane collaboration tool, and data synthesis and subgroup analyses were performed using the Review Manager program.

**Results:** Six studies involving 707 participants were included in the meta-analysis. XYS significantly improved TCE compared to WM (RR = 1.15, 95% CI: 1.05, 1.26, *p* = 0.002) with high heterogeneity (*I* ^*2*^ = 59%, *p* = 0.06). Combination therapy also showed higher TCE than WM alone (RR = 1.22, 95% CI: 1.05, 1.41, *p* = 0.008), and the heterogeneity was low (*I* ^*2*^ = 0%, *p* = 0.86). The results showed a greater reduction in DSS in the XYS and combination therapy groups than in the WM alone group (SMD = −0.72, 95% CI: −0.90, −0.53, *p* < 0.00001) with low heterogeneity (*I* ^*2*^ = 44%, *p* = 0.15), especially for abdominal distension and upper abdominal pain. AEs occurred less frequently in the XYS and combination therapy groups than in the WM alone group (RR = 0.20, 95% CI: 0.07, 0.63, *p* = 0.006), and the heterogeneity was low (*I* ^*2*^ = 45%, *p* = 0.18). The certainty of the evidence for each outcome was rated from “very low” to “high.”

**Conclusion:** This review suggests that XYS is effective and safe for reducing complaints in patients with FD. However, high-quality RCTs should be conducted to establish more convincing therapeutic evidence of XYS for the treatment of FD.

**Systematic Review Registration:** https://www.crd.york.ac.uk/prospero, CRD42020178842

## 1 Introduction

Functional dyspepsia (FD) is a common disease with a worldwide prevalence of 5%–11% (Ford et al., 2015). One or more symptoms, including postprandial fullness, early satiety, epigastric pain, and burning, can be present in patients with FD (Stanghellini et al., 2016). According to the main symptoms of patients, FD can be divided into two categories: postprandial distress syndrome (PDS) and epigastric pain syndrome (EPS). (Vanheel et al., 2017). The pathophysiology of FD is unclear; however, it is known to be related to gastric motility disorders, visceral hypersensitivity, and psychosocial dysfunction (Ly et al., 2015).

Although the details of clinical practice guidelines vary by region, prokinetics (PK), anti-secretory drugs (AS), antidepressants (AD), and *Helicobacter pylori* eradication therapy are the standard treatments for FD. However, since conventional medicine is not sufficiently effective and often exhibits adverse effects, more than 50% of patients with FD seek complementary and alternative medicine, such as acupuncture and herbal treatment (Ganguli et al., 2004). Herbal medicines are composed of various physiologically active compounds, among which certain substances exert therapeutic effects through synergistic interactions (Kim et al., 2015). Therefore, it can be effective in treating diseases such as FD, which have complex etiologies (Chiarioni et al., 2018).

*Xiaoyao-san* (XYS; also called *Soyo-san* in traditional Korean medicine and *Shoyo-san* in Kampo medicine) is a traditional herbal formula composed of eight botanical drugs: *Glycyrrhizae radix et rhizoma*, *Angelicae sinensis radix*, *Poria cocos*, *Paeoniae radix alba*, *Atractylodis macrocephalae rhizoma*, *Bupleuri radix*, *Menthae herba*, and *Zingiberis rhizoma recens* (Qin et al., 2009). XYS is one of the representative prescriptions traditionally used for gynecological diseases such as menopausal symptoms (Chen et al., 2011; Lee et al., 2021). Some laboratory studies have demonstrated estrogen- (Hu et al., 2022) and antidepressant-like effects of XYS (Dai et al., 2010; Yuan et al., 2020). At the same time, XYS has been commonly prescribed for FD patients, especially with *Disharmony of liver and spleen systems pattern*. This pattern of patients is characterized by complaints of dyspeptic symptoms aggravated by emotional factors and accompanied by symptoms of overall deficiency such as fatigue and general weakness (Huang et al., 1992; Ren et al., 2006).

Two previous reviews evaluated the effect of XYS on FD: one compared XYS with PK alone (Qin et al., 2009), and the other analyzed the therapeutic effect of XYS monotherapy (Tang et al., 2019). Both studies had some limitations in that the comparators and outcome variables were limited, which made it difficult to generalize the results. Therefore, more clinical studies evaluating various outcome measures should be conducted to establish objective evidence for XYS as an alternative treatment for FD. This review aimed to assess the efficacy and safety of XYS for FD by comparing it with Western medicine (WM). In addition, the effect of combination therapy (XYS plus WM) was evaluated and compared with that of WM alone.

## 2 Materials and methods

XYS contains the following botanical drugs in varying proportions: the dried root of Bupleurum chinese DC [Apiaceae; Bupleuri radix]; the dried root of Angelica sinensis (Oliv.) Diels [Apiaceae; Angelicae sinensis radix]; the dried root Paeonia lactiflora Pall [Paeoniaceae; Paeoniae radix alba]; the dried rhizome of Atractylodes macrocephala Koidz [Compositae; Atractylodis macrocephalae rhizoma]; the dried sclerotium of Poria cocos (Schw.) Wolf [Polyporaceae; Poria cocos]; the dried rhizome of Zingiber officinale Roscoe [Zingiberaceae; Zingiberis rhizoma recens]; the dried aerial parts of Mentha canadensis L [Lamiaceae; Menthae herba]; the dried root and rhizome of Glycyrrhiza uralensis Fisch [Leguminosae; Glycyrrhizae radix et rhizoma].

The protocol for this systematic review was registered with PROSPERO (No. CRD42020178842) (Ha et al., 2020). This study was conducted according to the guidelines of the Preferred Reporting Items for Systematic Reviews and Meta-analysis (PRISMA) (Page et al., 2021) (Supplementary Table S1).

### 2.1 Inclusion and exclusion criteria

#### 2.1.1 Types of patients

Patients diagnosed with FD according to the ROME criteria, an international diagnostic tool for functional gastrointestinal disorders (FGID), were included with no restrictions on region, sex, or age. The ROME criteria were first published in 1994 and underwent four revisions until 2016. Therefore, studies conducted before 1994 were only included if the symptoms mentioned in the paper were judged to meet the ROME criteria by the consensus of two independent researchers (HL and HJ). Disagreements regarding eligibility for inclusion were arbitrated by a third party (N-YH). Secondary dyspeptic symptoms caused by diseases such as gastric ulcers and stomach cancer were excluded.

#### 2.1.2 Types of interventions

Studies on XYS, modified XYS, and combination therapy (co-administration of XYS and WM) were included in this review. Modified XYS refers to XYS prescriptions with additional botanical drugs, such as *Danzhi*-XYS (DZXYS), in which the dried root bark of Paeonia *×* suffruticosa Andrews [Paeoniaceae; Moutan cortex] and the dried ripe fruit of Gardenia jasminoides J. Ellis [Rubiaceae; Gardeniae fructus] are added to XYS. Clinical trials without details of interventions, such as drug dose, dosage form, and route of administration, were not covered in this review to ensure accuracy and reproducibility. Studies involving other alternative therapies, including acupuncture and moxibustion, were excluded.

#### 2.1.3 Types of comparators

This review included the following comparative studies:

First, XYS was compared with the placebo, no-treatment, and WM groups, including PK, AS, and AD.

Second, combination therapy (XYS plus WM) was compared with WM alone.

#### 2.1.4 Types of outcomes

The primary outcome variable was the total clinical efficacy rate (TCE).

The secondary outcome variables included dyspepsia-related symptom score (DSS), Hamilton Anxiety Rating Scale (HAM-A), Hamilton Depression Rating Scale (HAM-D), recurrence rate after treatment, and incidence of adverse events (AEs).

#### 2.1.5 Types of study designs

Randomized controlled trials (RCTs) were included. Animal studies, cell experiments, and case reports were excluded.

### 2.2 Search strategy

A literature search was conducted using the following databases up to 31 January 2023: Embase, Medline, Allied and Complementary Medicine Database, and Cochrane Central Register of Controlled Trials for global databases; Korean Medical Database, KoreaMed, Korean Studies Information Service System, National Digital Science Library, Oriental Medicine Advanced Searching Integrated System, China National Knowledge Infrastructure Database, and Citation Information by Nii for Asian databases.

Symptom-related keywords such as “dyspepsia,” “indigestion,” “pain,” “distress,” and “discomfort” and intervention-related keywords such as “*Soyo*,” “*Xiaoyao*,” and “*Shoyo*” were merged to form a search formula (Table 1). The detailed searching strategies for all databases were presented in the Supplementary Table S2. No language restrictions were imposed.

**TABLE 1:** Search strategy used in Medline *via* PubMed.

No	Search items
#1	indigestion*
#2	Intestin* OR Digest* OR Gastr* OR gut OR epigastr* OR stomach*
#3	#1 AND #2
#4	dyspepsia*
#5	epigastric [tiab] AND pain [tiab]
#6	epigastric [tiab] AND burn* [tiab]
#7	Rome* AND criteria*
#8	(disturbance* OR disorder* OR difficult* OR dysfunction* OR disease* OR impair* OR condition* OR abnormal* OR illness* OR patholog* OR discomfort* OR hazard* OR damage* OR injur* OR irritab* OR pain* OR distress* OR burning) AND postprandial*
#9	#3 OR #4 OR #5 OR #6 OR #7 OR #8
#10	Herbal medicine [MeSH Terms]
#11	Plants, medicinal [MeSH Terms]
#12	Medicine, traditional [MeSH Terms]
#13	Drugs, Chinese herbal [MeSH Terms]
#14	Herb* [tiab]
#15	Plant [tiab] OR plants [tiab]
#16	Phytomedicine [tiab]
#17	Botanical [tiab]
#18	Weed* [tiab]
#19	Algae [tiab]
#20	Fungi [tiab] OR fungus [tiab]
#21	(Traditional [tiab] OR Chinese [tiab] OR herbal [tiab]) AND medicine [tiab]
#22	(Oriental [tiab] OR Chinese [tiab]) AND tradition* [tiab]
#23	#10 OR #11 OR #12 OR #13 OR #14 OR #15 OR #16 OR #17 OR #18 OR #19 OR #20 OR #21 OR #22
#24	Soyo* OR Shoyo* OR Xiaoyao* OR Xiao yao* OR Shiauyau* OR Shiau yau*
#25	Randomized controlled trial [pt]
#26	Controlled clinical trial [pt]
#27	Randomized [tiab]
#28	Randomly [tiab]
#29	Trial [ti]
#30	#25 OR #26 OR #27 OR #28 OR #29
#31	#9 AND #23 AND #24 AND #30

### 2.3 Study selection and extraction

Two independent researchers (HL and HJ) collected the studies using Endnote X9 (Clarivate Analytics, Philadelphia), reviewed the title, abstract, and entire original text, and included relevant studies according to the inclusion and exclusion criteria. In the event of disagreement between the two reviewers regarding eligibility, they reviewed the article together to reach a consensus. If necessary, a third reviewer (N-YH) functioned as an arbitrator. After the entire text of the selected studies was confirmed, study information, including the first author, publication year, country, diagnostic criteria, interventions, treatment duration, results, and adverse events, was extracted and tabulated using Microsoft Excel (2019).

### 2.4 Assessment of risk of bias

Two reviewers (HL and HJ) independently assessed the risk of bias using the Cochrane Collaboration tool (Higgins et al., 2022). Seven items were evaluated: random sequence generation, allocation concealment, blinding of participants and personnel, blinding of outcome assessment, incomplete outcome data, selective reporting, and other bias. Each item was classified into three categories of risk of bias: “high,” “unclear,” and “low.” If the opinions of the two researchers differed, they attempted to reach a consensus through discussion by consulting a third reviewer (N-YH).

### 2.5 Data analysis and synthesis

Statistical analyses were performed using the Review Manager program (RevMan, computer program, Version 5.4.1, The Cochrane Collaboration, 2020). Quantitative data from two or more studies was integrated using a synthesis for the same outcome measures; if it was not appropriate, a qualitative description of each study’s data was presented in a tabulation format. To measure the effect size, the risk ratio (RR) and 95% confidence interval (CI) were used for dichotomous data, such as TCE and incidence of AEs, whereas the mean difference (MD) and 95% CI were evaluated for continuous data, such as DSS. The *I* ^*2*^ statistic was used to assess the heterogeneity of the included studies. The random-effects model was used for the meta-analysis when it was judged that significant heterogeneity existed (*I* ^*2*^ value of greater than 50%); otherwise, the fixed-effects model was used. For each outcome measure, forest plots were presented to display the estimated effect sizes from the included studies.

### 2.6 Subgroup and sensitivity analyses

Subgroup analysis was performed with a sufficient number of studies according to the type of intervention and treatment duration to adjust for heterogeneity. Sensitivity analyses were conducted to determine the robustness of the outcomes and other sources of heterogeneity.

### 2.7 Publication bias

A funnel plot was used to evaluate publication bias if more than ten studies were included in the analysis.

### 2.8 Evaluation of the certainty of the evidence

The Grading of Recommendations Assessment, Development, and Evaluation (GRADE) tool was used to evaluate the quality of cumulative evidence in this review (Goldet and Howick, 2013). By assessing the factors such as the risk of bias, inconsistency, and indirectness, the certainty level of evidence was judged using four categories of “very low,” “low,” “moderate,” and “high.”

## 3 Results

### 3.1 Study selection

**FIGURE 1:**
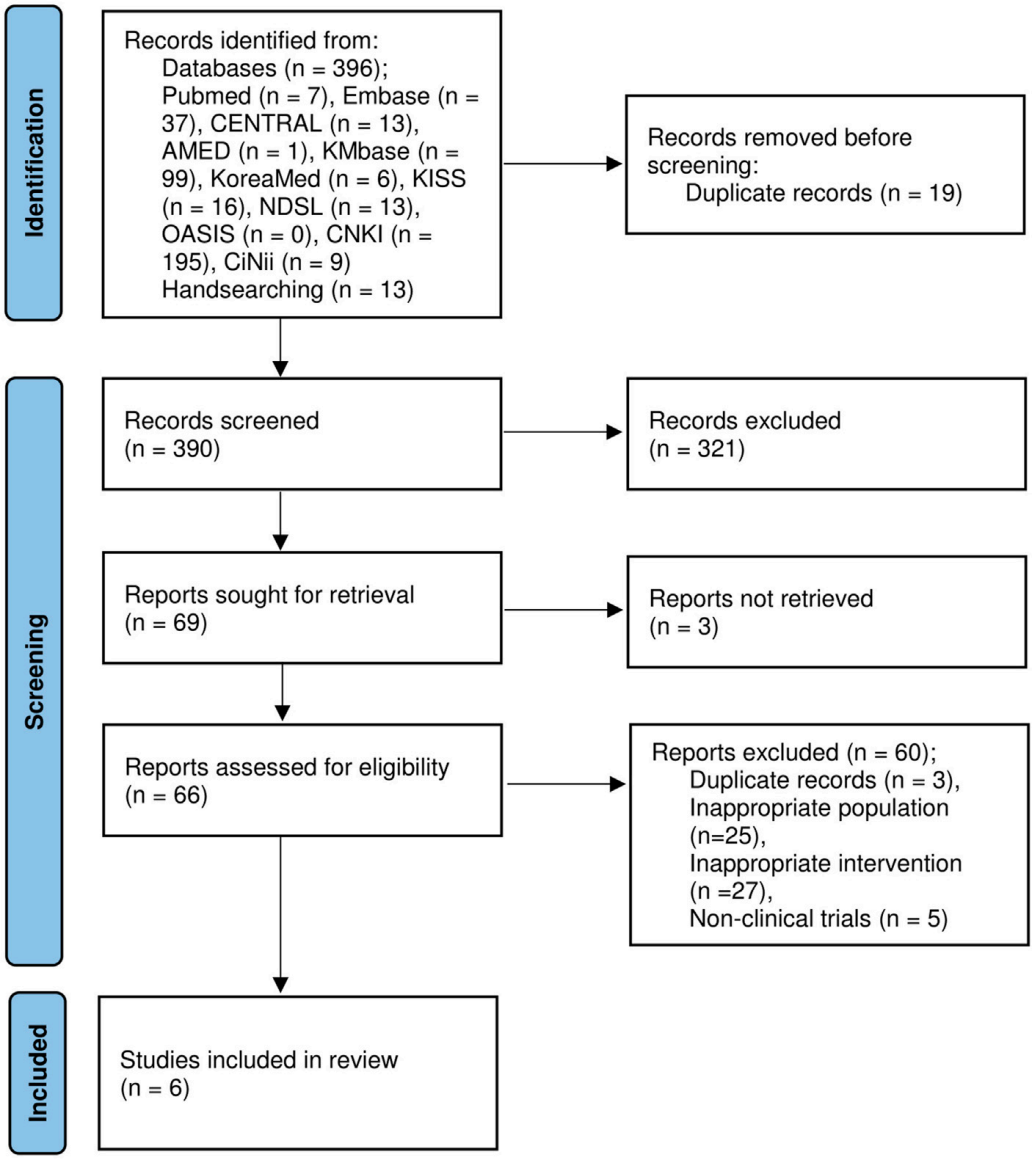
Flow chart of the literature search.

As of 31 January 2023, 396 articles were retrieved from 11 databases, and 13 articles were identified through manual searches. After excluding 19 overlapping articles, 390 were selected. After screening the titles and abstracts of the included articles, 321 were excluded because they did not meet the inclusion criteria. Among the 66 full texts retrieved, 3 studies were duplicate records, 25 were not involved in the relevant population, 27 did not use an appropriate intervention, and 5 were non-clinical trials. Finally, six studies (Li, 2006; Li et al., 2006; Xu, 2007; Wang, 2014; Wu, 2014; Liu and Xie, 2017) including 707 subjects were included in this systematic review (Figure 1) (Page et al., 2021).

### 3.2 Characteristics of included studies

All six included articles involving 707 patients were published in China between 2006 and 2017 (Table 2). For the study design, five (Li, 2006; Xu, 2007; Wang, 2014; Wu, 2014; Liu and Xie, 2017) were two-arm, parallel-design RCTs, and one (Li et al., 2006) was a three-arm trial comparing XYS, WM, and combination therapy. Among the five trials, three (Xu, 2007; Wang, 2014; Wu, 2014) compared XYS with WM, and two (Li, 2006; Liu and Xie, 2017) compared combination therapy with WM alone. Regarding the type of XYS, four studies (Xu, 2007; Wang, 2014; Wu, 2014; Liu and Xie, 2017) used XYS, and two (Li, 2006; Li et al., 2006) used DZXYS in the intervention group. Herbal prescriptions and their components in the included RCTs are presented in Table 3. Regarding the type of WM, two studies (Li et al., 2006; Wu, 2014) used Mosapride, and two (Xu, 2007; Wang, 2014) used Domperidone plus enzyme compounds in the control group to compare XYS with WM. In comparing the combination therapy and WM groups, one study (Li, 2006) compared XYS plus Tibolone *versus* Mosapride plus Tibolone, and the other (Liu and Xie, 2017) compared XYS plus AD *versus* Mosapride plus AD. The number of patients in the included studies ranged from 80 to 209, with an average of 118, and the trial duration ranged from 2 weeks to 2 months, with an average of 4 weeks. All studies used the XYS decoction as the dosage form. As for the diagnostic criteria for FD, three studies adopted Rome II (Li, 2006; Li et al., 2006; Xu, 2007), and three adopted Rome III (Wang, 2014; Wu, 2014; Liu and Xie, 2017). Five trials used XYS in the treatment of FD according to pattern identification (PI), including *Disharmony of liver and spleen systems pattern* (Li, 2006; Li et al., 2006; Liu and Xie, 2017), *Disharmony of liver and stomach systems pattern* (Wang, 2014), and *Tangled cold and heat pattern* (Xu, 2007).

**TABLE 2:** Characteristics of included studies.

Study ID	Country	Diagnostic criteria/Pattern identification	Sample size (E/C)	Male to female ratio (E/C)	Age (years) (m ± sd) (E/C)	Experimental (E)	Control (C)	Duration (follow- up)	Outcome measures	Adverse events
Li et al. (2006)	China	Rome II/*Disharmony of liver and spleen systems pattern*	114 (30:39:45)	0:114 [Perimenopausal females]	NR	DZXYS (bid)	(1) Mosapride (5 mg, 1T tid) (2) DZXYS (bid) + Tibolone (2.5 mg, 1T qd)	4 weeks (−)	① TCE	Mild headache, dizziness, insomnia
Wang (2014)	China	Rome III/*Disharmony of liver and stomach systems pattern*	92 (52/40)	20:32/16:24	40.3 ± 9.8/41.2 ± 10.0	XYS (qd)	Domperidone (10 mg, 1T tid) + CDEC (1C tid)	2 weeks (−)	① TCE	NR
Wu (2014)	China	Rome III/-	92 (46/46)	21:25/20:26	51.23 ± 14.21/52.39 ± 14.65	XYS (bid)	Mosapride (5 mg, 1T tid)	2 months (−)	① TCE ② DSS ③ GECE	E (1): mild diarrhea (1)/C (11): dizziness (7), mildly elevated transaminases (4)
Xu (2007)	China	Rome II/*Tangled cold and heat pattern*	209 (106/103)	54:52/51:52	37.2 ± 10.5/39.3 ± 9.2	XYS (qd)	Domperidone (10 mg, 1T tid) + MET (3T tid)	2 weeks (−)	① TCE ② DSS	NR
Li (2006)	China	Rome II/*Disharmony of liver and spleen systems pattern*	80 (50/30)	0:80 [Perimenopausal females]	49.62 ± 2.097/48.07 ± 3.050	DZXYS (bid) + Tibolone (2.5 mg, 1T qd)	Mosapride (5 mg, 1T tid) + Tibolone (2.5 mg, 1T qd)	4 weeks (6 months)	① TCE ② DSS ③ R6MAT ④ FSH⑤ LH ⑦ FSH/LH	E (3): increased stool frequency (3)/C (4): mild diarrhea (2), mild headache and dizziness (1), insomnia (1)
Liu and Xie (2017)	China	ROME III/*Disharmony of liver and spleen systems pattern*	120 (60/60)	18:42/21:39	48.9 ± 14.5/50.8 ± 13.8	XYS (bid) + Flupentixol/Melitracen (0.5 mg/10 mg, 1T bid)	Mosapride (5 mg, 1T tid) + Flupentixol/Melitracen (0.5 mg/10 mg, 1T bid)	4 weeks (−)	① TCE ② DSS ③ HAM-D ④ HAM-A	NR

Bid, Bis in die (= twice a day); CDEC, compound digestive enzyme capsule; DSS, Dyspepsia-related symptom score; DZXYS, *Danzhi-Xiaoyao-san*; E2, estradiol; FSH, Follicle-stimulating hormone; GECE, gastric emptying clinical efficacy; HAM-A, hamilton anxiety rating scale; HAM-D, hamilton depression rating scale; LH, luteinizing hormone; MET, multienzyme tablet; NR, not reported; Qd, Quaque die (= once a day); R6MAT, Recurrence 6 months after treatment; T, tablet; TCE, total clinical efficacy rate; XYS, *Xiaoyao-san*.

**TABLE 3:** Herbal prescription and its components in the included RCTs.

Study ID	Prescription (volume per intake, frequency); extraction process	Medical institution	Species name, daily dosage	Quality control	Chemical profile
Li et al. (2006)	Danzhi-Xiaoyao-san (NR,bid); decoction from mixtures of botanical drugs	Hubei college of traditional Chinese medicine, Wuhan, China	Dried root of *Bupleurum chinense* DC., 15 g	NR	NR
			Dried root of *Angelica sinensis* (Oliv.) Diels, 15 g		
			Dried root of *Paeoniae lactiflora* Pall., 15 g		
			Dried rhizome of *Atractylodis macrocephalae* Koidz., 15 g		
			Dried sclerotium of *Poria cocos* (Schw.) Wolf., 15 g		
			Dried root bark of *Paeonia suffruticosa* Andrews, 12 g		
			Dried ripe fruit of *Gardenia jasminoides* J.Ellis, 12 g		
			Roasted rhizome of *Zingiber officinale* Roscoe, 6 g		
			Dried aerial parts of *Mentha canadensis* L., 3 g		
			Dried root and rhizome of *Glycyrrhizae uralensis* Fisch., 9 g		
Wang (2014)	Xiaoyao-san (NR,NR); decoction from mixtures of botanical drugs	Tianjin Hedong hospital of traditional Chinese medicine, Tianjin, China	Dried root of *Bupleurum chinense* DC., 12 g	NR	NR
			Dried root of *Angelica sinensis* (Oliv.) Diels, 12 g		
			Dried root of *Paeoniae lactiflora* Pall., 12 g		
			Dried rhizome of *Atractylodis macrocephalae* Koidz., 12 g		
			Dried sclerotium of *Poria cocos* (Schw.) Wolf., 12 g		
			Processed rhizome of *Zingiber officinale* Roscoe, 3 g		
			Dried aerial parts of *Mentha canadensis* L., 3 g		
			Dried root and rhizome of *Glycyrrhizae uralensis* Fisch., 6 g		
Wu (2014)	Xiaoyao-san (200 mL,bid); decoction from mixtures of botanical drugs	NR	Dried root of *Bupleurum chinense* DC., 10 g	NR	NR
			Dried root of *Angelica sinensis* (Oliv.) Diels, 6 g		
			Dried root of *Paeoniae lactiflora* Pall., 15 g		
			Stir-baked rhizome of *Atractylodis macrocephalae* Koidz., 15 g		
			Dried sclerotium of *Poria cocos* (Schw.) Wolf., 15 g		
			Roasted rhizome of *Zingiber officinale* Roscoe, 6 g		
			Dried aerial parts of *Mentha canadensis* L., 6 g		
			Stir-baked root and rhizome of *Glycyrrhizae uralensis* Fisch., 6 g		
Xu (2007)	Xiaoyao-san (NR,NR); decoction from mixtures of botanical drugs	West China hospital, Sichuan university, Chengdu, China	Dried root of *Bupleurum chinense* DC., 10 g	NR	NR
			Dried root of *Angelica sinensis* (Oliv.) Diels, 15 g		
			Dried root of *Paeoniae lactiflora* Pall., 15 g		
			Dried rhizome of *Atractylodis macrocephalae* Koidz., 12 g		
			Dried sclerotium of *Poria cocos* (Schw.) Wolf., 12 g		
			Dried rhizome of *Zingiber officinale* Roscoe, 3 g		
			Dried aerial parts of *Mentha canadensis* L., 3 g		
			Dried root and rhizome of *Glycyrrhizae uralensis* Fisch., 6 g		
Li (2006)	Danzhi-Xiaoyao-san (100 mL,bid); decoction from mixtures of botanical drugs	Hubei Zhongyi college affiliated hospital, Hubei, China; Wuhan union hospital, Hubei, China	Dried root of *Bupleurum chinense* DC., 15 g	NR	NR
			Dried root of *Angelica sinensis* (Oliv.) Diels, 15 g		
			Dried root of *Paeoniae lactiflora* Pall., 15 g		
			Dried rhizome of *Atractylodis macrocephalae* Koidz., 15 g		
			Dried sclerotium of *Poria cocos* (Schw.) Wolf., 15 g		
			Dried root bark of *Paeonia suffruticosa* Andrews, 12 g		
			Dried ripe fruit of *Gardenia jasminoides* J.Ellis, 12 g		
			Roasted rhizome of *Zingiber officinale* Roscoe, 6 g		
			Dried aerial parts of *Mentha canadensis* L., 3 g		
			Dried root and rhizome of *Glycyrrhizae uralensis* Fisch., 9 g		
Liu and Xie (2017)	Xiaoyao-san (NR,bid); decoction from mixtures of botanical drugs	NR	Dried root of *Bupleurum chinense* DC., 15 g	NR	NR
			Dried root of *Angelica sinensis* (Oliv.) Diels, 15 g		
			Dried root of *Paeoniae lactiflora* Pall., 12 g		
			Dried rhizome of *Atractylodis macrocephalae* Koidz., 12 g		
			Dried sclerotium of *Poria cocos* (Schw.) Wolf., 15 g		
			Dried rhizome of *Zingiber officinale* Roscoe, 10 g		
			Dried aerial parts of *Mentha canadensis* L., 6 g		
			Stir-baked root and rhizome of *Glycyrrhizae uralensis* Fisch., 6 g		

Bid, Bis in die (= twice a day); NR, not reported.

### 3.3 Risk of bias assessment

**FIGURE 2:**
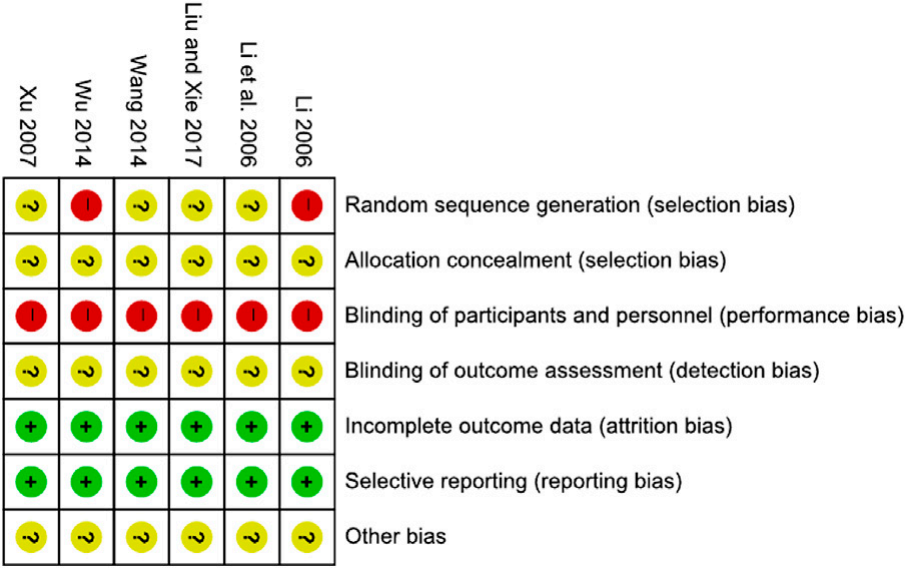
Summary of the risk of bias for each included study.

**FIGURE 3:**
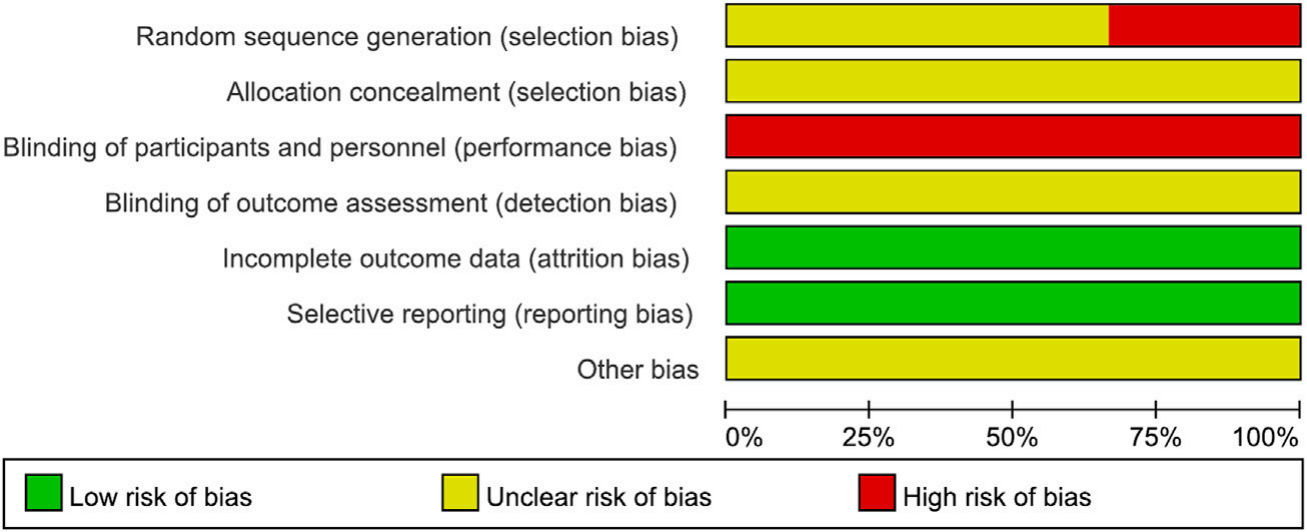
Graph showing the risk of bias across all included studies.

The risk of bias is presented in Figures 2, 3.

#### 3.3.1 Random sequence generation

Two studies (Li, 2006; Wu, 2014) that assigned subjects according to the visit order were evaluated as having a high risk of bias. The remaining four studies that did not describe the method of random sequence generation were assessed as having an unclear risk of bias.

#### 3.3.2 Allocation concealment

All six studies that did not describe the method of concealment were judged to have an unclear risk of bias.

#### 3.3.3 Blinding of the participants and personnel

All six studies, in which two interventions with different appearances between the XYS decoction and the WM were used without any blinding, were evaluated to have a high risk of bias.

#### 3.3.4 Blinding of outcome assessment

The risk of bias in all six studies with no explanation of blinding for outcome assessment was deemed questionable.

#### 3.3.5 Incomplete outcome data

All six trials were evaluated as having a low risk of bias because no missing or incomplete outcomes were identified.

#### 3.3.6 Selective reporting

All six studies reported the results mentioned in the Methods section and were assessed to have a low risk of bias.

#### 3.3.7 Other bias

Because there was insufficient evidence to identify additional forms of bias, all six studies were judged to have an uncertain risk of bias.

### 3.4 Meta-analysis results

#### 3.4.1 Primary outcome: Total clinical efficacy rate

In this review, TCE was used to compare the therapeutic effects of XYS and WM in FD. The TCE revealed the number of patients who demonstrated effective results in terms of dyspeptic symptoms improvement.

**FIGURE 4:**
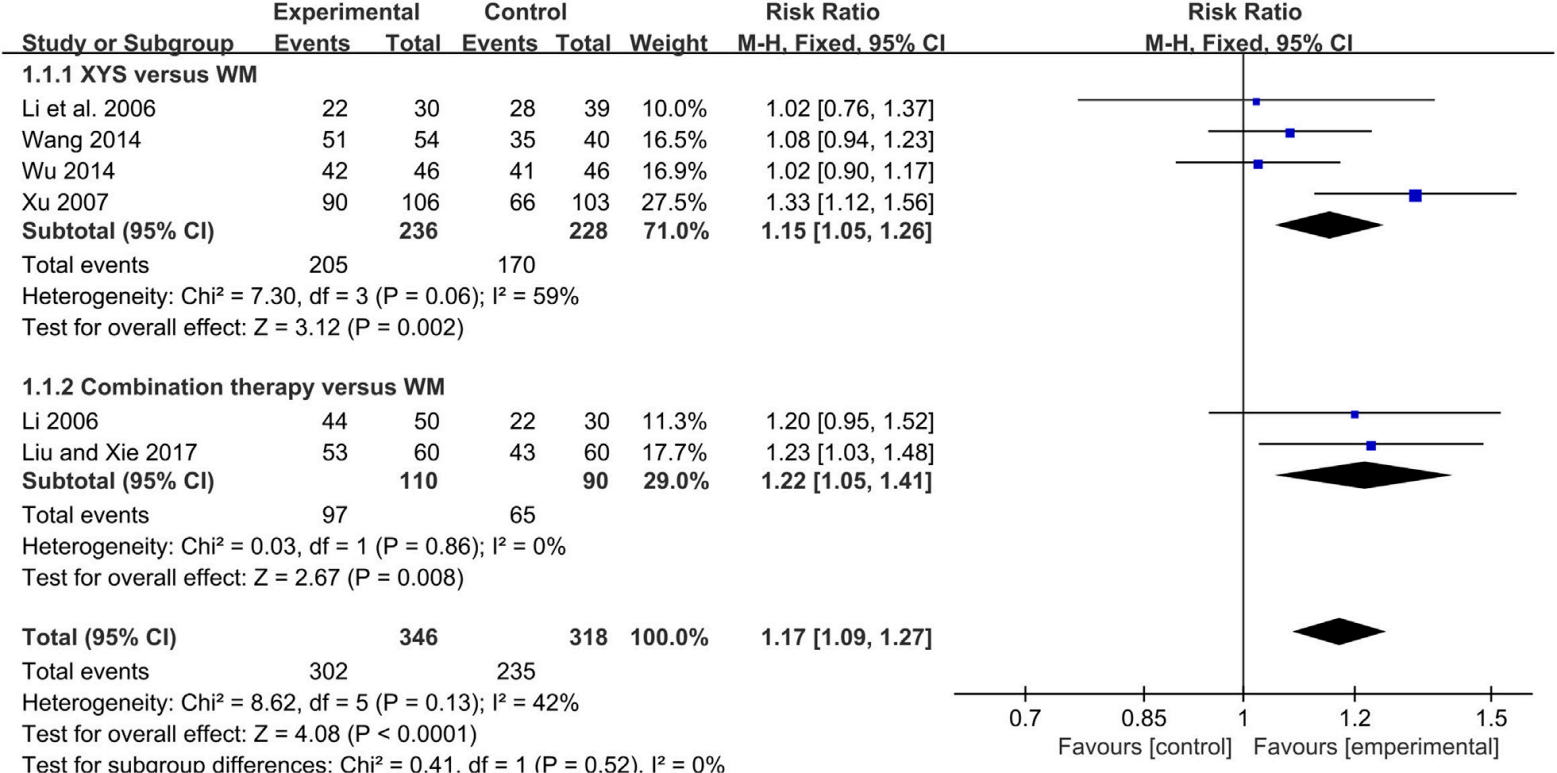
Forest plot of the comparison for total clinical efficacy rate between the XYS and WM groups.

A total of 664 subjects from six RCTs were included in the meta-analysis (Li, 2006; Li et al., 2006; Xu, 2007; Wang, 2014; Wu, 2014; Liu and Xie, 2017). The experimental group showed a higher TCE than the control group (RR = 1.17, 95% CI: 1.09, 1.27, *p* < 0.0001), and the heterogeneity was low (*I* ^*2*^ = 42%, *p* = 0.13). Depending on the type of intervention used in the experimental group, the studies were subdivided into two groups: 1) XYS and 2) combination therapy. In an analysis of 464 patients in four studies (Li et al., 2006; Xu, 2007; Wang, 2014; Wu, 2014), XYS showed significantly higher TCE than WM (RR = 1.15, 95% CI: 1.05, 1.26, *p* = 0.002) with high heterogeneity (*I* ^*2*^ = 59%, *p* = 0.06). Two studies including 200 participants (Li, 2006; Liu and Xie, 2017) reported that combination therapy also showed higher TCE than WM alone (RR = 1.22, 95% CI: 1.05, 1.41, *p* = 0.008) with low heterogeneity (*I* ^*2*^ = 0%, *p* = 0.86) (Figure 4).

#### 3.4.2 Secondary outcomes: Dyspepsia-related symptom score

**FIGURE 5:**
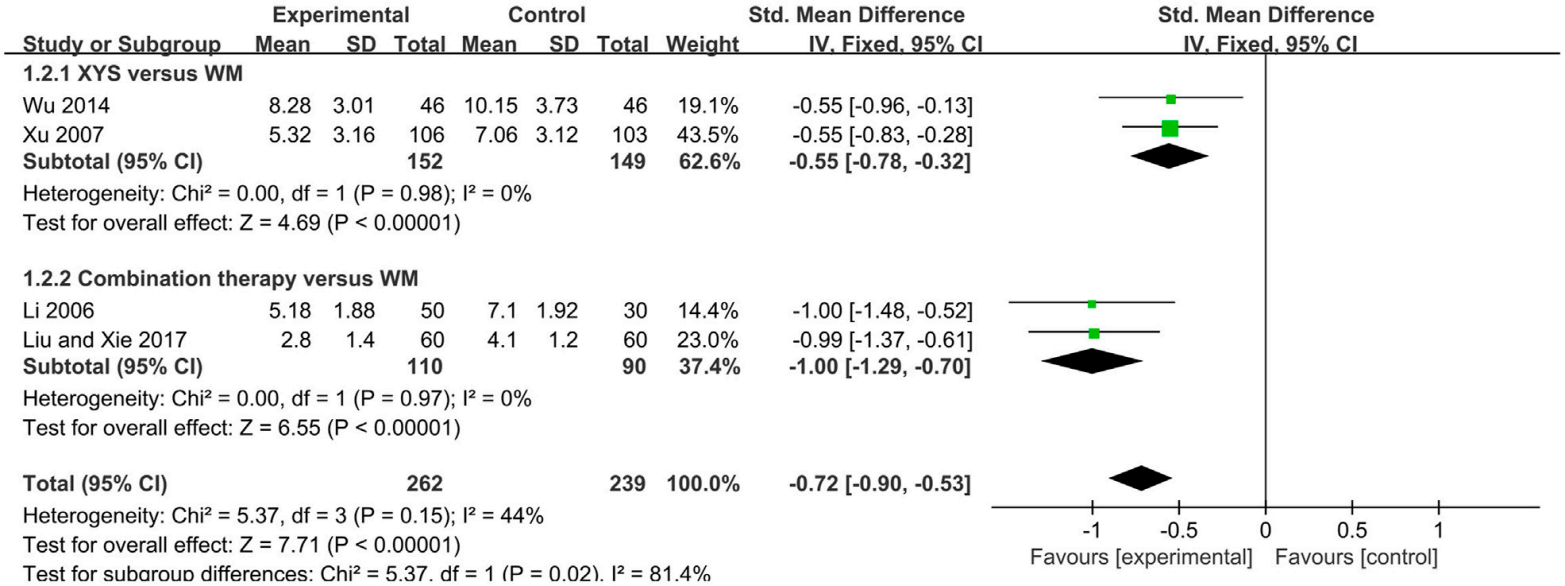
Forest plot of the comparison for dyspepsia-related symptom score between the XYS and WM groups.

DSS is a scale used to evaluate the severity of dyspeptic symptoms. Four studies with 501 participants (Li, 2006; Xu, 2007; Wu, 2014; Liu and Xie, 2017) were included in the meta-analysis. As a result, the experimental group significantly relieved DSS compared with the control group (standardized mean difference [SMD] = −0.72, 95% CI: −0.90, −0.53, *p* < 0.00001), and the heterogeneity was low (*I* ^*2*^ = 44%, *p* = 0.15). According to the type of intervention used in the experimental group, the trials were subdivided into two groups: 1) XYS and 2) combination therapy. Analysis of two studies including 301 participants (Xu, 2007; Wu, 2014), XYS significantly improved dyspeptic symptoms compared with WM (SMD = −0.55, 95% CI: −0.78, −0.32, *p* < 0.00001) with low heterogeneity (*I* ^*2*^ = 0%, *p* = 0.98). Of the 200 patients in the two articles (Li, 2006; Liu and Xie, 2017), DSS was significantly lower in the combination therapy group than in the WM alone group (SMD = −1.00, 95% CI: −1.29, −0.70, *p* < 0.00001) with low heterogeneity (*I* ^*2*^ = 0%, *p* = 0.97) (Figure 5).

For specific dyspeptic symptoms, two trials (Li, 2006; Wu, 2014) compared the efficacy of XYS and WM groups on abdominal distension, upper abdominal pain, belching, and early satiety; one study (Wu, 2014) compared XYS and Mosapride, and the other (Li, 2006) compared XYS combined with Tibolone *versus* Mosapride combined with Tibolone.

##### 3.4.2.1 Abdominal distension

**FIGURE 6:**
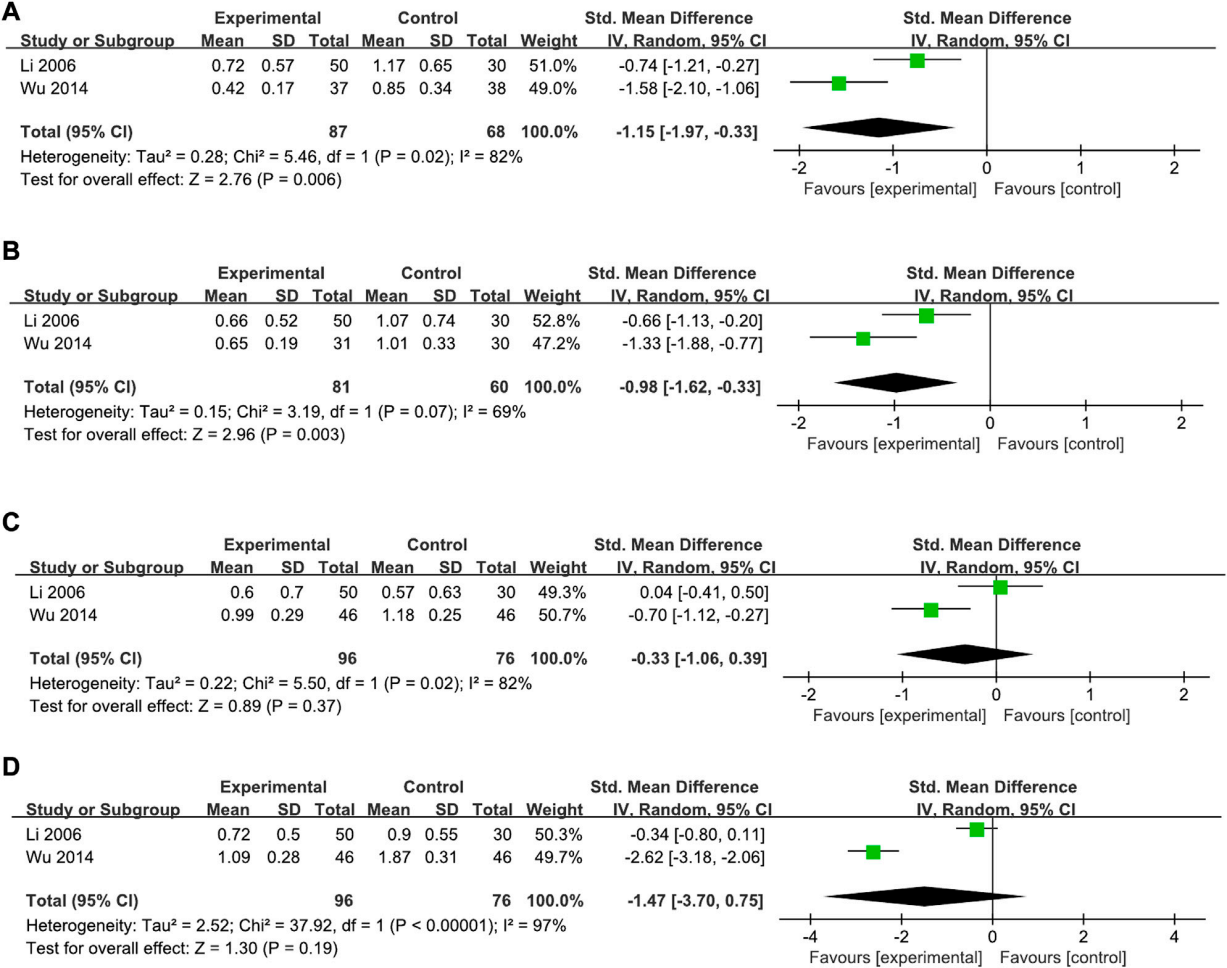
Forest plot of the comparison for the scores of specific dyspeptic symptoms between the XYS and WM groups. **(A)** Abdominal distension; **(B)** upper abdominal pain; **(C)** belching; and **(D)** early satiety.

A total of 155 cases in the two studies showed a significant improvement in abdominal distension in the XYS group compared to the WM group (SMD = −1.15, 95% CI: −1.97, −0.33, *p* = 0.006), with high heterogeneity (*I* ^*2*^ = 82%, *p* = 0.02) (Figure 6A).

##### 3.4.2.2 Upper abdominal pain

A total of 141 cases in two studies reported a significant improvement in upper abdominal pain in the XYS group compared to the WM group (SMD = −0.98, 95% CI: −1.62, −0.33, *p* = 0.003), with high heterogeneity (*I* ^*2*^ = 69%, *p* = 0.07) (Figure 6B).

##### 3.4.2.3 Belching

A total of 172 cases in two studies showed no significant differences were found between the two groups on the symptom of belching (SMD = −0.33, 95% CI: −1.06, 0.39, *p* = 0.37), with high heterogeneity (*I* ^*2*^ = 82%, *p* = 0.02) (Figure 6C).

##### 3.4.2.4 Early satiety

A total of 172 cases in two studies reported no significant differences in the symptoms of early satiety between the two groups (SMD = −1.47, 95% CI: −3.70, 0.75, *p* = 0.19), with high heterogeneity (*I* ^*2*^ = 97%, *p* < 0.00001) (Figure 6D).

#### 3.4.3 Subgroup analysis

**FIGURE 7:**
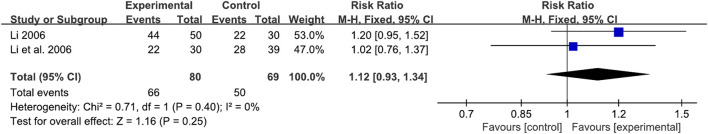
Forest plot of the comparison for total clinical efficacy rate between the DZXYZ and WM groups.

In this review, both the XYS and DZXYS were integrated into the XYS group. Thus, an additional subgroup analysis was performed based on the comparison of the DZXYS and WM groups to minimize heterogeneity. One study (Li et al., 2006) compared DZXYS and Mosapride, and the other (Li, 2006) compared DZXYS plus Tibolone *versus* Mosapride plus Tibolone. A total of 172 perimenopausal patients with FD in two RCTs showed no significant difference in TCE in the DZXYS *versus* WM groups (RR = 1.12, 95% CI: 0.93, 1.34, *p* = 0.25) and the heterogeneity was low (*I* ^*2*^ = 0%, *p* = 0.40) (Figure 7).

### 3.5 Evaluation of adverse events

**FIGURE 8:**
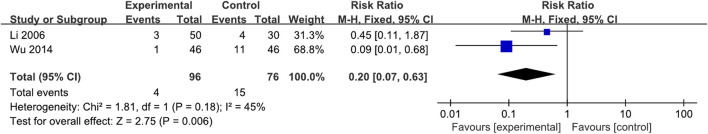
Forest plot of the comparison for the incidence of adverse events between the XYS and WM groups.

Of the six studies, three mentioned AEs; one study listed only the types of AEs, regardless of the interventions, while two reported detailed information. Li et al. (2006) reported mild headache, dizziness, and insomnia during the study period. Wu (2014) reported that mild diarrhea occurred in the XYS group and dizziness and mildly elevated transaminases occurred in the Mosapride group. Li (2006) reported that increased stool frequency occurred in the DZXYS combined with Tibolone group and mild diarrhea, mild headache and dizziness, and insomnia in the Mosapride combined with Tibolone group. A meta-analysis of two studies showed that there were significantly fewer AEs in the experimental group (XYS and combination therapy) than in the control group (WM alone) (RR = 0.20, 95% CI: 0.07, 0.63, *p* = 0.006), with low heterogeneity (*I* ^*2*^ = 45%, *p* = 0.18) (Figure 8).

### 3.6 Assessment of publication bias

Because each synthesis contained no more than 10 articles, publication bias was not analyzed using a funnel plot in this review.

### 3.7 Grading the quality of evidence

The certainty of the meta-evidence for the efficacy of XYS for the patients with FD was “high” on AEs; “moderate” on TCE, DSS, and upper abdominal pain; “low” on abdominal distension; and “very low” on belching and early satiety (Table 4). The risk of bias in all included studies was judged serious, as a high-performance bias was observed. The high heterogeneity across studies and the imprecision of the results in meta-analyses have resulted in a downgraded level of evidence.

**TABLE 4:** GRADE evidence profile.

Outcome (no. of studies)	Quality assessment					No. Of patients		RR/SMD (95% CI)	Certainty	Importance
	Risk of bias	Inconsistency	Indirectness	Imprecision	Other considerations	XYS combination therapy	WM			
TCE (6 RCTs)	Serious^a^	Not serious	Not serious	Not serious	None	302/346 (87.3%)	235/318 (73.9%)	RR = 1.17 (1.09, 1.27)	⊕⊕⊕○	CRITICAL
									Moderate	
DSS (4 RCTs)	Serious^a^	Not serious	Not serious	Not serious	None	262	239	SMD = −0.72 (−0.90, −0.53)	⊕⊕⊕○	CRITICAL
									Moderate	
Abdominal distension (2 RCTs)	Serious^a^	Serious^b^	Not serious	Not serious	None	87	68	SMD = −1.15 (−1.97, −0.33)	⊕⊕○○	CRITICAL
									Low	
Upper abdominal pain (2 RCTs)	Serious^a^	Not serious	Not serious	Not serious	None	81	60	SMD = −0.98 (−1.62, −0.33)	⊕⊕⊕○	CRITICAL
									Moderate	
Belching (2 RCTs)	Serious^a^	Serious^b^	Not serious	Serious^c^	None	96	76	SMD = −0.33 (−1.06, 0.39)	⊕○○○	CRITICAL
									Very low	
Early satiety (2 RCTs)	Serious^a^	Serious^b^	Not serious	Serious^c^	None	96	76	SMD = −1.47 (−3.70, 0.75)	⊕○○○	CRITICAL
									Very low	
Adverse events (2 RCTs)	Serious^a^	Not serious	Not serious	Not serious	Strong association^d^	4/96 (4.2%)	15/76 (19.7%)	RR = 0.20 (0.07, 0.63)	⊕⊕⊕⊕	CRITICAL
									High	

CI, confidence interval; DSS, Dyspepsia-related symptom score; RCT, randomized controlled trial; RR, risk ratio; SMD, standardized mean difference; TCE, total clinical efficacy rate; WM, western medicine; XYS, *Xiaoyao-san*.

^a^ Most studies had an unclear risk of selection and detection biases. Performance bias was high in the trials. Therefore, the evidence was downgraded by one level

^b^ The results were inconsistent across studies (I^2^ > 75%). Therefore, the evidence was downgraded by one level.

^c^ The 95% confidence interval overlapped with no effect. Therefore, the evidence was downgraded by one level.

^d^ The effect was large (RR < 0.5). Therefore, the evidence was upgraded by one level.

## 4 Discussion

According to the ROME IV criteria, if one or more of the following symptoms (postprandial fullness, early satiety, epigastric pain, or burning sensation) starts at least 6 months before the diagnosis and appears in the last 3 months, the patient is diagnosed with FD (Stanghellini et al., 2016). FD is classified into two subtypes, PDS and EPS, based on the patient’s main complaint (Vanheel et al., 2017). As the distribution of FD subtypes varies by region, the treatment guidelines for FD also differ between countries (Oh and Kwon, 2019). According to the clinical guidelines in the United States and Canada, if there is no response to *H. pylori* eradication therapy or proton pump inhibitors (PPIs), tricyclic antidepressants (TCAs) are recommended, followed by PK (Moayyedi et al., 2017). This is because TCA is more effective than PK in the United States and Canada, where the proportion of EPS subtypes is relatively high. In contrast, in Korea, there are many cases of PDS or overlap subtypes (Oh and Kwon, 2019). Therefore, recent clinical practice guidelines for FD in Korea recommend that PK and PPI be used primarily in patients with FD (Oh et al., 2020). Despite these conventional treatments, symptoms resolve in only half of FD patients, who are at risk of relapse or the development of other FGID (Olafsdottir et al., 2012; Talley and Ford, 2015).

### 4.1 Summary of the evidence

In this study, a systematic literature review and meta-analysis was performed to evaluate the efficacy and safety of XYS as a monotherapy and as an adjuvant treatment to conventional WM for FD. Six RCTs with 707 patients were included in this study. As critical outcomes, the TCE and DSS results showed a significant improvement in the XYS and combination therapy groups compared to the WM alone group. In particular, for the symptoms of abdominal distension and upper abdominal pain, XYS significantly alleviated the severity of symptoms compared with Mosapride when used alone or in combination with Tibolone. The above two symptoms are common in FD, which suggests that XYS can be used as an alternative therapy for FD patients who show an insufficient response to conventional WM treatment such as PK or prefer traditional medicine. Additionally, fewer adverse events were reported in the XYS group than in the WM group.

To specifically identify the effects of XYS, the subgroup analysis was performed involving the administration of DZXYS. As a result, DZXYS monotherapy and in combination with Tibolone may be an option for perimenopausal individuals with FD whose symptoms are poorly managed by customary treatments and are prone to recurrence.

FD is the most common FGID and is related to abnormal neuromodulation of the brain-gut axis, disharmony in the gut microbiome, and an imbalance of gastrointestinal hormones (Mertz, 2003; Fukui et al., 2018). In traditional Chinese medicine (TCM), XYS is used for dyspeptic symptoms, including stiffness or pain in the upper abdomen, reduced intake, and feelings of malaise or tiredness, which are caused by the pathophysiological pattern of *Disharmony of liver and spleen systems*. In TCM, XYS relieves these symptoms by circulating *qi*. In particular, the indication for XYS is closely related to *Liver qi stagnation*, expressed as emotional instability that can occur in conditions such as depressive disorders and postmenopausal syndrome (Lee and Jeong, 2017). In a meta-analysis of 26 studies with 1,837 patients with depression, combination therapy of XYS and AD significantly improved depressive symptoms compared to the AD alone group. These results suggest that XYS is clinically effective in relieving psychological symptoms such as anxiety and depression (Zhang et al., 2012). Recently, the antidepressant effect of XYS has been shown to be associated with brain-gut peptides (Liu et al., 2017), regulation of the gut microbiome (Zhu et al., 2019), inhibition of the hypothalamic-pituitary-adrenal axis (Zhu et al., 2014; Yan et al., 2018), and intracerebral interactions responsible for emotion and perception (Malagelada, 2020). The chemical compositions and pharmacological effects of the single components of XYS are presented in Supplementary Table S3. Among the six studies in this review, five performed PI before administration, which is defined as the process of determining the cause and nature of a patient’s disease in TCM (World Health Organization and Regional Office for the Western Pacific, 2007). Overall, *Disharmony of liver and spleen/stomach systems pattern*, which is related to the symptoms of indigestion accompanied by psychological depression, was the most frequently used pattern in the included studies. This suggests that XYS is recommended for FD patients with anxiety or depression by improving mental health.

### 4.2 Comparison with previous studies

Unlike previous reviews (Qin et al., 2009; Tang et al., 2019), this review analyzed the effect of XYS combined with WM and included various conventional WM, including PK, Tibolone, and AD. We only included studies that described herbal prescriptions in detail on administration information and components for clarity. In addition, the data search was performed comprehensively not only in China but also in global and Asian countries, including Japan and Korea, to reflect the results of clinical studies in countries with a high frequency of XYS administration for FD. Finally, unlike prior meta-analyses, our research demonstrated that DZXYS was not inferior to WM in the treatment of FD in perimenopausal females.

### 4.3 Limitations

This study has several limitations. First, there was a regional bias because all the studies in this review were based in China. Second, the quality of the included studies was low. The risk of bias in most of the studies were rated as “moderate” or “high” due to insufficient information on randomization process, allocation concealment, and methods of blinding outcome assessors. In addition, there were no previously published study protocols to assess other biases. In particular, double-blinding was not conducted in all of the included studies. When both herbal decoction and WM are used as interventions, it is difficult to perform blinding due to their differences in external characteristics. Therefore, in future studies, it is necessary to plan and adhere to a strict blinding protocol, such as using a double-placebo, and describe the method in detail. Third, during the selecting process, a significant number of studies that did not meet the intervention or diagnostic criteria were excluded from this review; thereafter, the number of included studies was insufficient to resolve the heterogeneities of intervention and treatment duration. Fourth, some meta-analyses were not available because studies that reported gastrointestinal hormones and gastric emptying rate or used validated questionnaires for dyspeptic or psychological symptoms were rare. There is no specific marker for FD and the patient complaints and symptoms are often ambiguous, making it difficult to quantify the improvement in dyspeptic symptoms. To resolve this ambiguity, various scales have been developed to measure the severity of FD symptoms. In this review, a meta-analysis of DSS showed that XYS significantly improved discomfort in the upper abdomen compared with WM. However, other variables could not be analyzed because no studies used standard questionnaires for FD, such as the Nepean Dyspepsia Index (Talley et al., 1999), the FD-related quality of life questionnaire (Lee et al., 2006), or the gastrointestinal symptom score (Adam et al., 2005). Further research using various validated outcome measures is required to compensate for these limitations. In addition, as for psychological symptoms, the results of analyzing HAM-A and HAM-D between the intervention and control groups were not reported due to lack of research. Fifth, the treatment duration and follow-up periods for assessing recurrence rates differed between studies, making it difficult to evaluate the persistent effect of XYS. Finally, the review found that quality control and chemical profile were not reported in any of the included studies, which has led to controversial conclusions. To address this issue, future clinical trials on botanical drug extracts should provide detailed information on the composition, extraction process, and drug extraction ratio of the study material. Additionally, presenting the basic pharmaceutical parameters of the extracts would allow for both qualitative and quantitative comparison.

### 4.4 Strengths and future perspectives

Compared to conventional WM, widely used to manage FD symptoms, both XYS and combination therapy showed significant efficacy and fewer adverse events. Thus, XYS may be administered to patients who do not respond to WM alone or who show adverse effects. Considering the low quality of the included studies, rigorous large-scale RCTs are required to confirm our findings. To strengthen the evidence for the effect of XYS in patients with FD, well-designed RCTs should be performed evaluating various outcome measures related to dyspepsia. Furthermore, future studies should assess the effectiveness of XYS with long-term follow-up.

## 5 Conclusion

By reviewing six RCTs with 707 participants, XYS monotherapy and combination therapy with WM can be considered effective and safe alternatives to WM in FD, relieving the severity of dyspeptic symptoms. However, the validity of the evidence based on included studies was disputed. Therefore, high-quality, double-blind RCTs are needed, with clear randomization and robust study methodology.

## Data Availability

The original contributions presented in the study are included in the article/Supplementary Material, further inquiries can be directed to the corresponding author.
