# Supplementary material for: Safety and efficacy of Xiaoyao-san for the treatment of functional dyspepsia: a systematic review and meta-analysis of randomized controlled trials
Source: Front Pharmacol. 2023 Apr 12;14:1114222. doi: 10.3389/fphar.2023.1114222 (PMC10130649; doi:10.3389/fphar.2023.1114222)
Supplement: Supplementary file 2 [file Table2.docx]

**Table S2. Search strategies for all databases.**

(1) Medline via PubMed

| **No.** | **Search items** |
| --- | --- |
| #1 | indigestion* |
| #2 | Intestin* OR Digest* OR Gastr* OR gut OR epigastr* OR stomach* |
| #3 | #1 AND #2 |
| #4 | dyspepsia* |
| #5 | epigastric [tiab] AND pain [tiab] |
| #6 | epigastric [tiab] AND burn* [tiab] |
| #7 | Rome* AND criteria* |
| #8 | (disturbance* OR disorder* OR difficult* OR dysfunction* OR disease* OR impair* OR condition* OR abnormal* OR illness* OR patholog* OR discomfort* OR hazard* OR damage* OR injur* OR irritab* OR pain* OR distress* OR burning) AND postprandial* |
| #9 | #3 OR #4 OR #5 OR #6 OR #7 OR #8 |
| #10 | Herbal medicine [MeSH Terms] |
| #11 | Plants, medicinal [MeSH Terms] |
| #12 | Medicine, traditional [MeSH Terms] |
| #13 | Drugs, Chinese herbal [MeSH Terms] |
| #14 | Herb* [tiab] |
| #15 | Plant [tiab] OR plants [tiab] |
| #16 | Phytomedicine [tiab] |
| #17 | Botanical [tiab] |
| #18 | Weed* [tiab] |
| #19 | Algae [tiab] |
| #20 | Fungi [tiab] OR fungus [tiab] |
| #21 | (Traditional [tiab] OR Chinese [tiab] OR herbal [tiab]) AND medicine [tiab] |
| #22 | (Oriental [tiab] OR Chinese [tiab]) AND tradition* [tiab] |
| #23 | #10 OR #11 OR #12 OR #13 OR #14 OR #15 OR #16 OR #17 OR #18 OR #19 OR #20 OR #21 OR #22 |
| #24 | Soyo* OR Shoyo* OR Xiaoyao* OR Xiao yao* OR Shiauyau* OR Shiau yau* |
| #25 | Randomized controlled trial [pt] |
| #26 | Controlled clinical trial [pt] |
| #27 | Randomized [tiab] |
| #28 | Randomly [tiab] |
| #29 | Trial [ti] |
| #30 | #25 OR #26 OR #27 OR #28 OR #29 |
| #31 | #9 AND #23 AND #24 AND #30 |

(2) Embase

| **No.** | **Search items** |
| --- | --- |
| #1 | indigestion* |
| #2 | Intestin* OR Digest* OR Gastr* OR gut OR epigastr* OR stomach* |
| #3 | #1 AND #2 |
| #4 | dyspepsia* |
| #5 | epigastric* AND pain* |
| #6 | epigastric* AND burn* |
| #7 | Rome* AND criteria* |
| #8 | (disturbance* OR disorder* OR difficult* OR dysfunction* OR disease* OR impair* OR condition* OR abnormal* OR illness* OR patholog* OR discomfort* OR hazard* OR damage* OR injur* OR irritab* OR pain* OR distress* OR burning) AND postprandial* |
| #9 | #3 OR #4 OR #5 OR #6 OR #7 OR #8 |
| #10 | 'plant extract'/exp OR 'traditional medicine'/exp OR 'alternative medicine'/exp |
| #11 | 'plant extract':ab,ti |
| #12 | 'phytotherapy'/exp |
| #13 | (herbal NEAR/1 medic*):ab,ti |
| #14 | (medicinal NEAR/1 herb*):ab,ti |
| #15 | (chinese NEAR/1 herb*):ab,ti |
| #16 | (chinese NEAR/1 medic*):ab,ti |
| #17 | (korea* NEAR/1 medic*):ab,ti |
| #18 | (oriental NEAR/1 medic*):ab,ti |
| #19 | (kampo NEAR/1 medic*):ab,ti |
| #20 | (japan* NEAR/1 medic*):ab,ti |
| #21 | #10 OR #11 OR #12 OR #13 OR #14 OR #15 OR #16 OR #17 OR #18 OR #19 OR #20 |
| #22 | Soyo* |
| #23 | Shoyo* |
| #24 | Xiaoyao* |
| #25 | 'Xiao yao*' |
| #26 | Shiauyau* |
| #27 | 'Shiau yau*' |
| #28 | #22 OR #23 OR #24 OR #25 OR #26 OR #27 |
| #29 | #9 AND #21 AND #28 |

(3) Allied and Complementary Medicine Database

| **No.** | **Search items** |
| --- | --- |
| S1 | indigestion* |
| S2 | Intestin* or Digest* or Gastr* or gut or epigastr* or stomach* |
| S3 | S1 AND S2 |
| S4 | dyspepsia* OR Indigest* |
| S5 | epigastric* AND pain* |
| S6 | epigastric* AND burn* |
| S7 | Rome* AND criteria* |
| S8 | (disturbance* or disorder* or difficult* or dysfunction* or disease* or impair* or condition* or abnormal* or illness* or patholog* or discomfort* or hazard* or damage* or injur* or irritab* or pain* or distress* or burning) AND postprandial* |
| S9 | S3 OR S4 OR S5 OR S6 OR S7 OR S8 |
| S10 | (DE "PHYTOTHERAPY") OR (DE "DRUGS CHINESE HERBAL") OR (DE "PLANTS MEDICINAL") OR (DE "HERBAL DRUGS") OR (TI Chinese N3 medic*) OR (AB Chinese N3 medic*) OR (TI Chinese N3 herb*) OR (AB Chinese N3 herb*) OR (TI Chinese N3 drug*) OR (AB Chinese N3 drug*) OR (TI Chinese N3 formul*) OR (AB Chinese N3 formul*) OR (TI Chinese N3 plant*) OR (AB Chinese N3 plant*) OR (TI Chinese N3 prescri*) OR (AB Chinese N3 prescri*) OR (TI phyto N6 drug*) OR (AB phyto N6 drug*) OR (TI phyto N6 therap*) OR (AB phyto N6 therap*) OR (TI phyto N6 treatment*) OR (AB phyto N6 treatment*) OR (TI phyto N6 medicin*) OR (AB phyto N6 medicin*) OR (TI complementary N3 therap*) OR (AB complementary N3 therap*) OR (TI complementary N3 medicin*) OR (AB complementary N3 medicin*) OR (TI complementary N3 treatment*) OR (AB complementary N3 treatment*) OR (TI alternativ* N3 therap*) OR (AB alternativ* N3 therap*) OR (TI alternativ* N3 medicin*) OR (AB alternativ* N3 medicin*) OR (TI alternativ* N3 treatment*) OR (AB alternativ* N3 treatment*) OR (TI plant* N6 medicine*) OR (AB plant* N6 medicine*) |
| S11 | Soyo* OR Shoyo* OR Xiaoyao* OR Xiao yao* OR Shiauyau* OR Shiau yau* |
| S12 | S9 AND S10 AND S11 |

(4) Cochrane Central Register of Controlled Trials

| **No.** | **Search items** |
| --- | --- |
| #1 | indigestion.mp. |
| #2 | (Intestin$ or Digest$ or Gastr$ or gut or epigastr$ or stomach$).mp. |
| #3 | #1 AND #2 |
| #4 | dyspepsia$ |
| #5 | (epigastric adj2 pain).ti,ab. |
| #6 | (epigastric adj2 burn$).mp. |
| #7 | (Rome adj2 criteria).mp. |
| #8 | ((disturbance$ or disorder$ or difficult$ or dysfunction$ or disease$ or impair$ or condition$ or abnormal$ or illness$ or patholog$ or discomfort$ or hazard$ or damage$ or injur$ or irritab$ or pain$ or distress$ or burning) adj2 postprandial).mp. |
| #9 | #3 OR #4 OR #5 OR #6 OR #7 OR #8 |
| #10 | MeSH descriptor: [Phytotherapy] explode all trees |
| #11 | MeSH descriptor: [Medicine, Traditional] explode all trees |
| #12 | MeSH descriptor: [Plants, Medicinal] explode all trees |
| #13 | MeSH descriptor: [Medicine, East Asian Traditional] explode all trees |
| #14 | MeSH descriptor: [Herbal Medicine] explode all trees |
| #15 | MeSH descriptor: [Drugs, Chinese Herbal] explode all tree |
| #16 | Chinese near/3 medic* |
| #17 | Chinese near/3 herb* |
| #18 | Chinese near/3 drug* |
| #19 | Chinese near/3 formul* |
| #20 | Chinese near/3 plant* |
| #21 | Chinese near/3 prescri* |
| #22 | Phyto near/6 drug* |
| #23 | Phyto near/6 therap* |
| #24 | Phyto near/6 treatment* |
| #25 | Phyto near/6 medicin* |
| #26 | Complementary near/3 therap* |
| #27 | Complementary near/3 medicin* |
| #28 | Complementary near/3 treatment* |
| #29 | Alternativ* near/3 therap* |
| #30 | Alternativ* near/3 medicin* |
| #31 | Alternativ* near/3 treatment* |
| #32 | plant* near/6 medicine* |
| #33 | #10 or #11 or #12 or #13 or #14 or #15 or #16 or #17 or #18 or #19 or #20 or #21 or #22 or #23 or #24 or #25 or #26 or #27 or #28 or #29 or #30 or #31 or #32 |
| #34 | Soyo* |
| #35 | Shoyo* |
| #36 | Xiaoyao* |
| #37 | Xiao yao* |
| #38 | Shiauyau* |
| #39 | Shiau yau* |
| #40 | #34 OR #35 OR #36 OR #37 OR #38 OR #39 |
| #41 | #9 AND #33 AND #40 |

(5) Korean Medical Database

| **Search items** |
| --- |
| ([ALL=dyspepsia*] OR [ALL=Indigest*] OR [ALL=Intestin*] OR [ALL=Digest*] OR [ALL=Gastr*] OR [ALL=gut] OR [ALL=epigastr*] OR [ALL=stomach*] OR [ALL=Postprandial*] OR [ALL=소화] OR [ALL=복부] OR [ALL=식후] OR [ALL=속쓰림] OR [ALL=위장] OR [ALL=식욕] OR [ALL=위부] OR [ALL=복부] OR [ALL=복통] OR [ALL=구역] OR [ALL=구토] OR [ALL=포만])  AND ([ALL=소요*] OR [ALL=Soyo*]) |

(6) KoreaMed

| **No.** | **Search items** |
| --- | --- |
| #1 | dyspepsia* OR Indigest* OR Intestin* OR Digest* OR Gastr* OR gut OR epigastr* OR stomach* OR Postprandial* |
| #2 | Soyo* OR Shoyo* OR Xiaoyao* OR Xiao yao* |
| #3 | #1 AND #2 |

(7) Korean Studies Information Service System

| **Search items** |
| --- |
| (Dyspepsia OR Indigestion OR Gut OR 소화) AND (Soyo OR 소요) |

(8) National Digital Science Library

| **Search items** |
| --- |
| (dyspepsia* \| Indigest* \| Intestin* \| Digest* \| Gastr* \| gut \| epigastr* \| stomach* \| Postprandial* \| 소화 \| 복부 \| 식후 \| 속쓰림 \| 위장 \| 식욕 \| 위부 \| 복부 \| 복통 \| 구역 \| 구토 \| 포만)  AND (소요* \| Soyo* \| Shoyo* \| Xiaoyao* \| Xiao yao* \| Shiauyau* \| Shiau yau*) |

(9) Oriental Medicine Advanced Searching Integrated System

| **Search items** |
| --- |
| 소화불량 AND 소요 |

(10) China National Knowledge Infrastructure Database

| **Search items** |
| --- |
| (SU=消化不良+功能性消化不良+烧灼+食欲不振+上腹不适+上腹痛+腹胀+早饱+嗳气+dyspepsia+indigestion) AND (SU=Xiaoyao+Shiauyau+逍遥) |

(11) Citation Information by Nii

| **Search items** |
| --- |
| (消化不良 OR ディスペプシア OR 酸逆流 OR 灼熱感 OR 食思不振 OR 胃部不快感 OR 腹部不快感 OR 心窩部痛 OR 腹痛 OR 腹胀 OR 早期飽満感 OR 嘔気 OR 嘔吐 OR げっぷ OR 食後愁訴 OR 腹部愁訴 OR 消化器愁訴 OR 消化器症状 OR dyspepsia OR indigestion) AND (逍遥* OR Shoyo*) |
