# Supplementary material for: Safety and efficacy of Xiaoyao-san for the treatment of functional dyspepsia: a systematic review and meta-analysis of randomized controlled trials
Source: Front Pharmacol. 2023 Apr 12;14:1114222. doi: 10.3389/fphar.2023.1114222 (PMC10130649; doi:10.3389/fphar.2023.1114222)
Supplement: Supplementary file 3 [file Table3.docx]

**Table S3. Chemical compositions and pharmacological activities of the single components of *Xiaoyao-san*.**

| **Scientific name**  **(family)** | **Latin name** | **Chinese**  **name** | **Parts used** | **Chemical compositions** | **Pharmacological activities** |
| --- | --- | --- | --- | --- | --- |
| *Bupleurum chinese* DC.  (family *Apiaceae*) | *Bupleuri radix* | *Chai-Hu*  (柴胡) | Root | Saikosaponin a, Saikosaponin c, Saikosaponin d, Saikosaponin e, Prosaikogenin G, Prosaikogenin F, 2”-O-Acetylsaikosaponin a, 3”-O-Acetylsaikosaponin a, 6”-O-Acetylsaikosaponin a, 23-O-Acetylsaikosaponin a, 6”-O-Acetylsaikosaponin d, 23-Hydroxy-13𝛽, 28𝛽-epoxy-olean-11-ene-16-one 3-O-𝛽-D-glucopyranosyl-(1→3)-𝛽-D-fucopyranoside, 3𝛽,16𝛽-Dihydroxy-23-O-acetyl-13𝛽, 28𝛽-epoxy-olean-11-ene 3-O-𝛽-D-fucopyranoside, Bupleuroside I, Saikosaponin b_1_, Saikosaponin b_2_, 6”-O-Acetyl-saikosaponin b_2_, Saikosaponin h, Prosaikogenin D, Prosaikogenin A, 3𝛽,23,28-Trihydroxy-11, 13(18)-diene-16-one 3-O-𝛽-D-glucopyranosyl-(1→ 3)-𝛽-D-fucopyranoside, Bupleuroside V, Bupleuroside X, Bupleuroside XII, Saikosaponin v-1, Saikosaponin b_3_, Saikosaponin b_4_, Saikosaponin f, 3𝛽,16𝛽,23,28-Tetrahydroxy-11𝛼-methoxy-olean-12-ene 3-O-𝛽-D-fucopyranoside, 3𝛽,16𝛽,28-Trihydroxyl-11𝛼-methoxy-olean-12-ene-O-𝛽-D-fucopyranoside, Bupleuroside VII , Saikosaponin g, Saikosaponin i, Bupleuroside VIII, Bupleuroside XI, (2Z,8Z,10E)-pentadecatriene-4,6-diyne-1-ol, (2Z,8E,10E)-pentadecatriene-4,6-diyne-1-ol, (2Z,8Z,10E)-heptadecatriene-4,6-diyne-1-ol, Bupleurynol, Quercetin, Isorhamnetin, Isorhamnetin-3-O-glucoside, Puerarin, Rutin, Narcissin, Eugenin, Saikochrome A, Saikochromic acid, 7,4’-Dihydroxy-isoflavone-7-O-𝛽-D-glucoside, Saikochromoside A, Saikoisoflavonoside A, Nortrachelogenin, Nemerosin, Kaerophyllin, Isochaihulactone, Isokaerophyllin, (−)-yatein, Chinensinaphthol, Chaihunaphthone, Fumaric acid, Butanedioic acid, Pentadecanoic acid, Palmitoleic acid, Palmitic acid, Oleic acid, Stearic acid, 11-Hexadecenoic acid, 13-Octadecenoic acid, Linoleic acid, Tetracosanoic acid, 9S,12S,13S-Trihydroxy-10E-octadecenoic acid, 𝛼-Spinasterol, 24𝜉-Methylcholesta-7, 22E-diene-3𝛽,5𝛼,6𝛽-triol, 24𝜉-Ethylcholest-22E-end-3𝛽,5𝛼,6𝛽-Triol (Yang et al., 2017) | Antidepressant (Li et al., 2017; Wang et al., 2015), hepatoprotective (Li et al., 2015) |
| *Angelica sinensis* (Oliv.) Diels  (family *Apiaceae*) | *Angelicae sinensis radix* | *Dang-Gui*  (当归) | Root | ferulic acid, 3-butylphthalide, Z-butylidenephthalide, 3-butylidene-4-hydroxyphthalide, E-butylidenephthalide, senkyunolide A, Z-ligustilide, E-ligustilide, 6,7-epoxyligustilide, senkyunolide F, senkyunolide H, senkyunolide I, 6,7-dihydroxyligustilide (Lao et al., 2004) | Antidepressant (Gong et al., 2019), antispasmodic (Du et al., 2006), neuroprotective (Gong et al., 2016) |
| *Paeonia lactiflora* Pall. (family *Paeoniaceae*) | *Paeoniae radix alba* | *Bai-Shao*  (白芍) | Root | paeoniflorin sulfonate, tetragalloyl glucose, monoterpene, glycosides sulfite, paeoniflorin, albiflorin, oxypaeoniflorin, benzoylpaeoniflorin, gallolpaeoniflorin, galloylalbiflorin, 1’-O-galloyl sucrose, 6’-O-galloyl sucrose, 6-O-galloyl sucrose, benzoyloxypaeniflorin, paeonol, methyl gallate, gallic acid (Tan et al., 2020) | Anticonvulsant (Zhang et al., 1994), antidepressant (Jin et al., 2016; Wang et al., 2016), neuroprotective (Zhang et al., 2017) |
| *Atractylodes macrocephala* Koidz.  (family *Compositae*) | *Atractylodis macrocephalae*  *rhizoma* | *Bai-Zhu*  (白术) | Rhizome | atractylenolide III, atractylenolide IV, 3-acetyl-atractylon, β-eudesmol, biatractylenolide II (Zhu et al., 2021) | Improving disordered intestinal flora (Wang et al., 2014), inhibiting the gastrointestinal movement and spasm (Zhang et al., 1999), promoting the healing of intestinal mucosa injury (Song et al., 2014) |
| *Poria cocos* (Schw.) Wolf.  (family *Polyporaceae*) | *Poria cocos* | *Fu-Ling*  (茯苓) | Sclerotium | Pachymic acid, Tumulosic acid, Trametenolic acid, Eburicoic acid, 3-O-acetyl-16α-hydroxytrametenolic acid, 16α-Hydroxytrametenolic acid, O-acetylpachymic acid, O-acetylpachymic acid-25-ol, Methyl-O-acetylpachymate, Pachymic acid methyl ester, Ganoderic acid, 25-Hydroxypachymic acid, 25-Hydroxy-3-epitumulosic acid, 16α,25-Dihydroxyeburiconic acid, 16α-Hydroxyeburiconic acid, 3β,16α-Dihydroxy-7-oxo-24-methyllanosta-8,24(31)-dien-21-oic acid, 3α,16α-Dihydroxy-7-oxo-24-methyllanosta-8,24(31)-dien-21-oic acid, Oxotrametenolic acid, Acetyl eburicoic acid, Poricoic acid ZH, Poricoic acid ZU, Poricoic acid ZW, 3β,15α-Dihydroxy-24-oxolanosta-8-en- 21-oic acid, 3β-Acetyloxy-16α-hydroxy-24-oxolanost-8-en-21-oic acid, Daedaleanic acid B, 15α-Hydroxyeburiconic acid, 3α,16α,25-Trihydroxylanosta-8,24-dien- 21-oic acid, 16α,29-Dihydroxyeburiconic acid, 3β-Acetyloxy-16α,26-dihydroxylanosta-8,24-dien-21-oic acid, 15α-Hydroxy-3-oxolanosta-8,24-dien-21-oic acid, 16α-Hydroxy-3-oxolanosta-8,24-dien-21-oic acid, 3β,16α-Bis(acetyloxy)-29-hydroxylanosta-8,24-dien-21-oic acid, Hispindic acid B, 3β,15α-Bis(acetyloxy)-24- methylenelanost-8-en-21-oic acid, 16α-Acetyloxyeburiconic acid, 3-Epi-pachymic acid, Ceanphytamic acid A, Ceanphytamic acid B, Dehydrotrametenolic acid, Dehydropachymic acid, Dehydroeburicoic acid, 6α-Hydroxypolyporenic acid C, 3-Epi-dehydrotumulosic acid, 25-Hydroxy-3-epi-dehydrotumulosic acid, Dehydrotumulosic acid, Dehydroeburiconic acid, 3-O-Acetyl-16α-hydroxydehydrotrametenolic acid, 3-Epidehydropachymic acid, 3β,16α-Dihydroxylanosta-7,9(11),24-trien-21-oic acid, 6α-Hydroxydehydropachymic acid, 3β-p-Hydroxybenzoyldehydrotumulosic acid, 3β-Hydroxy-16α-acetoxy-lanosta-7,9(11),24-trien-21-oic acid, Polyporenic acid C, Dehydrotrametenonic acid, 15α-Hydroxydehydrotumulosic acid, 16α,25-Dihydroxydehydroeburicoic acid, 29-Hydroxypolyporenic acid C, Poriacosones A, Poriacosones B, 16α,27-Dihydroxydehydrotrametenoic acid, 3β,16α,30-Trihydroxy-24-methyllanosta-7,9(11),24(31)-trien-21-oic acid, 3β-Acetoxy-16α,24β-dihydroxylanosta-7,9(11),25-trien-21-oic acid, 29-Hydroxydehydrotumulosic acid, 29-Hydroxydehydropachymic acid, 3β,15α-Dihydroxylanosta-7,9(11),24-triene-21-oic acid, Dehydrosulphurenic acid, Dehydroeburicoic acid monoacetate, 3β-Acetoxylanosta-7,9(11),24-trien-21-oic acid, Poricoic acid ZE, Poricoic acid ZI, Poricoic acid ZL, Poricoic acid ZV, Coriacoic acid B, Coriacoic acid C, 6,16α-Dihydroxydehydrotrametenonic acid, 16α-Hydroxydehydrotrametenonic acid, 25,26-Dihydroxydehydropachymic acid, 3β,16α-Dihydroxy-24-hydroxymethyllanosta-7,9(11)-dien-21-oic acid, 15α-Hydroxydehydrotrametenolic acid, 16α-Hydroxydehydrotrametenoic acid, 16-Hydroxy-3,24-dioxolanosta-7,9(11)-dien-21-oic acid, 16α-Acetyloxy-24-methylene-3-oxolanosta-7,9(11)-dien-21-oic acid, Poricoic acid G, Poricoic acid H, 25-Hydroxyporicoic acid H, Poricoic acid GM, Poricoic acid HM, Poricoic acid GE, Poricoic acid ZA, Poricoic acid ZJ, Poricoic acid ZK, Poricoic acid ZR, 25-Methoxy-29-hydroxyporicoic acid HM, Poricoic acid A, Poricoic acid B, Poricoic acid C, Poricoic acid D, Poricoic acid DM, Poricoic acid AM, Poricoic acid E, Poricoic acid BM, Poricoic acid F, 16-Deoxyporicoic acid B, Poricoic acid CM, 25-Methoxyporicoic acid A, 26-Hydroxyporicoic acid DM, 25-Hydroxyporicoic acid C, Poricoic acid AE, Poricoic acid CE, 3,4-Secolanosta-4(28),7,9,24Z-tetraen-3,26-dioic acid, Poricoic acid BE, 16α-Hydroxy-3,4-secolanosta-4(28),7,9(11),24(31),25(27)-pentaene-3,21-dioic acid, Poricoic acid ZB, Poricoic acid ZC, Poricoic acid ZD, Poricoic acid ZG, Poricoic acid ZM, Poricoic acid ZO, Poricoic acid ZP, Poricoic acid ZN, Poricoic acid ZT, Poricoic acid ZQ, 5α,8α-Peroxydehydrotumulosic acid, 3-(2-Hydroxyacetoxy)-5α,8α-peroxydehydrotumulosic acid, Daedaleanic acid A, 11β-Ethoxydaedaleanic acid A, (3β,16α)-3-Acetyloxy-16-hydroxy-24-methylenelanosta-5,7(9),11-tetraene-21-oic acid, 16α-Hydroxy-3-oxo-24-methyllanosta-5,7,9(11),24(31)-tetraen-21-oic acid, 6,7-Dehydroporicoic acid H, Coriacoic acid A, Coriacoic acid D, β-Amyrin acetate, Oleanolic acid, 3-O-acetyloleanolic acid, 7-oxo-15-Hydroxydehydroabietic acid, Dehydroabietic acid methyl ester, Poricoic acid ZF, Dehydroabietic acid, 7-Oxocallitrisic acid, Pimaric acid, Ergosterol, (22E)-ergosta-5,7,9(11),22-tetraen-3β-ol, Ergosta-5,7-dien-3β-ol, (22E)-Ergosta-8(14),22-dien-3β-ol, (22E)-Ergosta-6,8(14),22-trien-3β-ol, (22E)-Ergosta-7,22-dien-3β-ol, Ergost-7-en-3β-ol, Ergosterol peroxide, Daucosterol, Cerevisterol, Biemnasterol, Β-Sitosterol, 3β,5α-Dihydroxy-ergosta-7,22-diene-6-one, 3β,5α,9α-Trihydroxy-ergosta-7,-dien-6-one, Ergosta-7,22-diene-3-one, 6,9-Epoxy-ergosta-7,22-diene-3-ol, Ergosta-4,22-diene-3-one, Ergosta-5,6-epoxy-7,22-dien-3-ol, Pregn-7-ene-2β,3α,15α,20-tetrol, Peroxy-ergosterol, Ergot sterone, 9,11-Dehydroergosterol peroxide, Pachyman, PolysaccharideH11, PC1, PC2, PC2-A, PC3, PC4, PCSC22, PCM1, PCM2, PCM3, PCM4, ac-PCM0, ac-PCM1, ac-PCM2, PCPWP, PCPWPS, ac-PCM3-I, ac-PCM3-II, ac-PCM4-I, ac-PCM4-II, wb-PCM1, wb-PCM2, wc-PCM1, wc-PCM2, wb-PCM3-I, wc-PCM3-I, wb-PCM3-II, wb-PCM4-I, wb, PCM4-II, wc-PCM3-II, wc-PCM4I, wc-PCM4-II, WIP, wb-PCM0, wc-PCM0, ab-PCM0, ab-PCM1, ab-PCM2-I, ab-PCM2-I, ab-PCM3-I, ab-PCM3-II, ab-PCM4-I, abPCM4-II, PCS1, PCS2, PCS3-I, PCS3-II, PCS4-I, PCS4-II, PC-PS, PCSG, Pi-PCM0, Pi-PCM1, Pi-PCM2, Pi-PCM3-I, Pi-PCM4-I, Pi-PCM3-II, Pi-PCM4-II, Polysaccharides from *Poria*, WSP, WSP-1, WSP-2, PCII, PCP, ATPCP, PCP-II, CMP33, PAC, FMGP, CMP3, PPS, CMP, PPSW-1, Sul-W-1, S-CMP, Polysaccharides (WRP), PPC, PCP-M, Sulfated pachymaran, PCWPW and PCWPS (Lu et al., 2021) | Antidepressant (Zhang et al., 2019), antispasmodic (Xiao et al., 2020), enteroprotective (Zou, 2019) |
| *Zingiber officinale* Roscoe  (family *Zingiberaceae*) | *Zingiberis rhizoma recens* | *Sheng-Jiao*  (生姜) | Rhizome | [6]-Paradol, [7]-Paradol, [8]-Paradol, [9]-Paradol, [10]-Paradol, [11]-Paradol, [13]-Paradol, Methyl [6]-paradol, [4]-Gingerol, [6]-Gingerol, [7]-Gingerol, [8]-Gingerol, [10]-Gingerol, Methyl [4]-gingerol, Methyl [6]-gingerol, [4]-Shogaol, [6]-Shogaol, [8]-Shogaol, [10]-Shogaol, [12]-Shogaol, Methyl [6]-shogaol, Methyl [8]-shogaol, Acetoxy-[4]-gingerol, Acetoxy-[6]-gingerol, Acetoxy-[8]-gingerol, Acetoxy-[10]-gingerol, Methyl acetoxy-[6]-gingerol, 1-Dehydro-[3]-gingerdione, 1-Dehydro-[6]-gingerdione, 1-Dehydro-[8]-gingerdione, 1-Dehydro-[10]-gingerdione, [4]-Gingerdiol, [6]-Gingerdiol, [8]-Gingerdiol, [10]-Gingerdiol, 5-Acetoxy-[4]-gingerdiol, 5-Acetoxy-[6]-gingerdiol, 5-Acetoxy-[7]-gingerdiol, Methyl 5-acetoxy-[4]-gingerdiol, Methyl 5-acetoxy-[6]-gingerdiol, Diacetoxy-[4]-gingerdiol, Diacetoxy-[6]-gingerdiol, Methyl diacetoxy-[4]-gingerdiol, Methyl diacetoxy-[6]-gingerdiol, Methyl diacetoxy-[10]-gingerdiol, 3-Dihydro-[6]-demethoxyshogaol, 5-Methoxy-[6]-gingerol, 1,7-bis-(4’-Hydroxy-3’-methoxyphenyl)-4-heptene-3-one, 1,7-bis-(4’-Hydroxy-3’-methoxyphenyl)-3,5-heptadione, 1-Dehydro-3-dihydro-[10]-gingerdione, 6-Dihydroparadol, Acetoxy-6-dihydroparadol, 1-(4’-Hydroxy-3’-methoxyphenyl)-7-octen-3-one, 1-(4’-Hydroxy-3’-methoxyphenyl)-7-decen-3-one, 1-(4’-Hydroxy-3’-methoxyphenyl)-7-dodecen-3-one, [4]-Isogingerol, 4-(4-Hydroxyphenyl)-2-butanone, 4-Hydroxy-3-methoxybenzenepropanal, 3,4-Dimethoxybenzenepropanal, Zingerone, Zingerone methyl ether, Gingerol, Zingerol 2-methyl ether (Jolad et al., 2004) | Antiserotonergic (Yamahara et al., 1989), chemopreventative (Mahady et al. 2003), enhancing gastric emptying (Yamahara et al., 1990) |
| *Mentha canadensis* L.  (family *Lamiaceae*) | *Menthae herba* | *Bo-He*  (薄荷) | Aerial parts | (4S)-7-hydroxy-carvone 7-O-β-D-glucopyranoside, (3β,11α)-3-hydroxy-11α-methoxy-olean-12-en-3-yl palmitate, (4R,6R)-carveol β-d-glucoside, (4R,6S)-carveol β-d-glucoside, (+)-neodihydrocarvy β-D-glucoside, (−)-dihydrocarvy β-D-glucoside, uroterpenol β-D-glucoside, spicatoside A, spicatoside B, (3S,6S)-cislinalool-3,7-oxide, (3R,9S)-megastigman-5-en-3,9-diol 3-O-β-d-glucopyranoside, linarionoside A, 1,1,5-trimethyl- 6-(3-hydroxyl) cyclohexene5-yl-1-β-D-pyranoglucoside, linarionoside B, (9S)-linarionoside B, (+)-jasmololone glycoside, (‒)-5′-(β-d-glucopyranosyloxy) jasmonic acid, maniladiol, 3β,28- dihydroxy-olean-12-enyl palmitate, olean-12-ene-28-arboxy-3-palmitate, ursolic acid, 1-(β-D-ribofuranosyl)-1H-1,2,4-triazone, naphthisoxazol A, menthalactone, 6-amino9-[1-(3,4-dihydroxy phenyl)ethyl]-9H-purine (He et al., 2019) | Regulating intestinal flora (Fang et al., 2022) |
| *Glycyrrhiza uralensis* Fisch.  (family *Leguminosae*) | *Glycyrrhizae radix et rhizoma* | *Gan-Cao*  (甘草) | Root and rhizome | glycyrrhizic acid, 22β-acetoxyl-glycyrrhizin, licorice-saponin E2, licorice-saponin G2, licorice-saponin A3, liquiritigenin, liquiritin, liquiritin apioside, isoliquiritigenin, isoliquiritin, isoliquiritin apioside, glabrone, licoisoflavone B, glycyrrhisoflavone, isoangustone A, licoflavone B, isolicoflavonol, licochalcone A, echinatin, licoagrochalcone C, glycyrol, glyasperin D, licoricidin, glycycoumarin, glabridin, licoisoflavanone (Shang et al., 2022; Song et al., 2017) | Antispasmodic (Sato et al., 2007), antiulcer (Dehpour et al., 1995) |

**References**

Dehpour, A. R., Zolfaghari, M. E., Samadian, T., Kobarfard, F., Faizi, M., and Assari, M. (1995). Antiulcer activities of liquorice and its derivatives in experimental gastric lesion induced by ibuprofen in rats. *Int. J. Pharm.* 119 (2), 133-138. doi: 10.1016/0378-5173(94)00377-H

Du, J., Bai, B., Kuang, X., Yu, Y., Wang, C., Ke, Y., et al. (2006). Ligustilide inhibits spontaneous and agonists- or K+ depolarization-induced contraction of rat uterus. *J. Ethnopharmacol.* 108 (1), 54-58. doi: 10.1016/j.jep.2006.04.011

Fang, C., Chen, G., Kan, J. (2022). Characterization and in vitro simulated gastrointestinal digestion and fermentation of Mentha haplocalyx polysaccharide. *Int. J. Biol. Macromol.* 222, 360-372. doi: org/10.1016/j.ijbiomac.2022.09.168

Gong, W., Zhou, Y., Li, X., Gao, X., Tian, J., Qin, X., et al. (2016). Neuroprotective and Cytotoxic Phthalides from Angelicae Sinensis Radix. *Molecules*. 21 (5). 549. doi: 10.3390/molecules21050549

Gong, W., Zhu, S., Chen, C., Yin, Q., Li, X., Du, G., et al. (2019). The Anti-depression Effect of Angelicae Sinensis Radix Is Related to the Pharmacological Activity of Modulating the Hematological Anomalies. *Front. Pharmacol.* 10, 192. doi: 10.3389/fphar.2019.00192

He, X. F., Geng, C. A., Huang, X. Y., Ma, Y. B., Zhang, X. M., Chen, J. J. (2019). Chemical Constituents from Mentha haplocalyx Briq. (Mentha canadensis L.) and Their α-Glucosidase Inhibitory Activities. *Nat. Prod. Bioprospect.* 9 (3), 223–229. doi: 10.1007/s13659-019-0207-0

Jin, Z. L., Gao, N., Xu, W., Xu, P., Li, S., Zheng, Y., et al. (2016). Receptor and transporter binding and activity profiles of albiflorin extracted from Radix paeoniae Alba. *Sci. Rep.* 6, 33793. doi: 10.1038/srep33793

Jolad, S. D., Lantz, R. C., Solyom, A. M., Chen, G. J., Bates, R. B., Timmermann, B. N. (2004). Fresh organically grown ginger (Zingiber officinale): composition and effects on LPS-induced PGE2 production. *Phytochemistry*. 65 (13), 1937-1954. doi: 10.1016/j.phytochem.2004.06.008

Lao, S. C., Li, S. P., Kan, Kelvin K. W., Li, P., Wan, J. B., Wang, Y. T., et al. (2004). Identification and quantification of 13 components in Angelica sinensis (Danggui) by gas chromatography–mass spectrometry coupled with pressurized liquid extraction. Analytica Chimica Acta. 526 (2), 131-137. doi: 10.1016/j.aca.2004.09.050

Li, Z. Y., Sun, H. M., Xing, J., Qin, X. M., and Du, G. H. (2015). Chemical and biological comparison of raw and vinegar-baked Radix Bupleuri. *J. Ethnopharmacol*. 165, 20-28. doi: 10.1016/j.jep.2015.02.024

Li, H. Y., Zhao, Y. H., Zeng, M. J., Fang, F., Li, M., Qin, T. T., et al. (2017) Saikosaponin D relieves unpredictable chronic mild stress induced depressive-like behavior in rats: involvement of HPA axis and hippocampal neurogenesis. *Psychopharmacology (Berl).* 234 (22), 3385-3394. doi: 10.1007/s00213-017-4720-8

Lu, J., Tian, J., Zhou, L., Meng, L., Chen, S., Ma, C., et al. (2021). Phytochemistry and Biological Activities of Poria. *J. Chem.* 2021, 6659775. doi: org/10.1155/2021/6659775

Mahady, G. B., Pendland, S. L., Yun, G. S., Lu, Z, Z., Stoia, A. (2003). Ginger (Zingiber officinale Roscoe) and the gingerols inhibit the growth of Cag A+ strains of Helicobacter pylori. *Anticancer Res*. 23 (5A), 3699-3702. https://www.ncbi.nlm.nih.gov/pmc/articles/PMC3761965/pdf/nihms123076.pdf

Sato, Y., He, J. X., Nagai, H., Tani, T., and Akao, T. (2007). Isoliquiritigenin, one of the antispasmodic principles of Glycyrrhiza Ularensis roots, acts in the lower part of intestine. *Biol. Pharm. Bull.* 30 (1), 145-149. doi: 10.1248/bpb.30.145

Shang, Z., Liu, C., Qiao, X., and Ye, M. (2022). Chemical analysis of the Chinese herbal medicine licorice (Gan-Cao): An update review*. J. Ethnopharmacol.* 299, 115686. doi: 10.1016/j.jep.2022.115686

Song, H. P., Li, R. L., Chen, X., Wang, Y. Y., Cai, J, Z., Liu, J, et al. (2014). Atractylodes macrocephala Koidz promotes intestinal epithelial restitution via the polyamine--voltage-gated K+ channel pathway. J. Ethnopharmacol. 152 (1), 163-172. doi: 10.1016/j.jep.2013.12.049

Song, W., Qiao, X., Chen, K., Wang, Y., Ji, S., Feng, J., et al. (2017). Biosynthesis-based quantitative analysis of 151 secondary metabolites of licorice to differentiate medicinal Glycyrrhiza specie and their hybrids. *Anal. Chem.* 89 (5), 3146–3153. doi: 10.1021/acs.analchem.6b04919

Tan, Y. Q., Chen, H. W., Li, J., and Wu, Q. J. (2020). Efficacy, Chemical Constituents, and Pharmacological Actions of Radix Paeoniae Rubra and Radix Paeoniae Alba. *Front. Pharmacol.* 11, 1054. doi: 10.3389/fphar.2020.01054

Wang, R., Zhou, G., Wang, M., Peng, Y., and Li, X. (2014). The Metabolism of Polysaccharide from Atractylodes macrocephala Koidz and Its Effect on Intestinal Microflora. *Evid. Based Complement. Alternat. Med.* 2014, 926381. doi: 10.1155/2014/926381

Wang, X., Feng, Q., Xiao, Y., and Li, P. (2015) Radix Bupleuri ameliorates depression by increasing nerve growth factor and brain-derived neurotrophic factor. *Int. J. Clin. Exp. Med*. 2015, 8 (6), 9205-9217. www.ijcem.com /ISSN:1940-5901/IJCEM0008403

Wang, Y. L., Wang, J. X., Hu, X. X., Chen, L., Qiu, Z. K., Zhao, N., et al. (2016). Antidepressant-like effects of albiflorin extracted from Radix paeoniae Alba. *J. Ethnopharmacol.* 179, 9–15. doi: 10.1016/j.jep.2015.12.029

Xiao, H. H., Zhang, M. B., Guo, Z. Q., and Yang, J. X. (2020). Study on mechanism poria in inhibiting contraction of small intestine in vitro. *Mod. Chin. Med*. 22 (9), 1478–1484. doi: 10.13313/j.issn.1673-4890.20191016010

Yamahara, J., Rong, H. Q., Iwamoto, M., Kobayashi, G., Matsuda, H., Fujimura, H. (1989). Active components of ginger exhibiting anti-serotinergic action. *Phytother. Res.* 3, 70-71. doi: 10.1002/ptr.2650030208

Yamahara, J., Huang, Q. R., Li, Y. H., Xu, L., Fujimura, H. (1990). Gastrointestinal motility enhancing effect of ginger and its active constituents. *Chem. Pharm. Bull. (Tokyo).* 38 (2), 430-431. doi: 10.1248/cpb.38.430

Yang, F., Dong, X., Yin, X., Wang, W., You, L., and Ni, J. (2017) Radix Bupleuri: A Review of Traditional Uses, Botany, Phytochemistry, Pharmacology, and Toxicology. *Biomed Res Int*. 2017, 7597596. doi: 10.1155/2017/7597596

Zhang, Y., Ming, L., Wang, Y., Ma, H. X., Ma, C. G., and Xu, S. Y. (1994). Anticonvulsant action of total glucosides of paeony root. *Chin. Pharm. Bull.* 05, 372–374. https://www.cnki.com.cn/Article/CJFDTOTAL-YAOL405.016.htm

Zhang, Y.Q., Xu, S.B., and Lin, Y.C. (1999) Gastrointestinal inhibitory effects of sesquiterpene lactones from Atractylodes macrocephala. *J. Chin. Med. Mater.* 12, 636–640. doi: 10.13863/j.issn1001-4454.1999.12.012

Zhang, Y., Qiao, L., Xu, W., Wang, X., Li, H., Xu, W., et al. (2017). Paeoniflorin attenuates cerebral ischemia-induced injury by regulating Ca2+/CaMKII/CREB signaling pathway. *Molecules.* 22 (3), 359. doi: 10.3390/molecules22030359

Zhang, J. Y., Tang, J., Zhang, Q., Wang, X. Q., and Li, X. W. (2019) Effects of sulfated pachymaran AMPA receptor expression in hippocampi of depression model rats. *Chin. J. Clin. Psychol*. 27 (6), 1086–1091. doi: 10.16128/j.cnki.1005-3611.2019.06.003

Zhu, Q., Lin, M., Zhuo, W., and Li, Y. (2021). Chemical Constituents from the Wild Atractylodes macrocephala Koidz and Acetylcholinesterase Inhibitory Activity Evaluation as Well as Molecular Docking Study. *Molecules.* 26 (23), 7299. doi: 10.3390/molecules26237299

Zou, Y. T. (2019) Characterization of Chemical Constituents of Poria cocos and Primary Study of their Protective Effects against Cisplatin-Induced Intestinal Injury. Master’s Theses, *Nanjing Univ. Chin. Med., Jiangsu, China*. https://cdmd.cnki.com.cn/Article/CDMD-10315-1019170271.htm
